# BIN1 protein isoforms are differentially expressed in astrocytes, neurons, and microglia: neuronal and astrocyte BIN1 are implicated in tau pathology

**DOI:** 10.1186/s13024-020-00387-3

**Published:** 2020-07-29

**Authors:** Mariko Taga, Vladislav A. Petyuk, Charles White, Galina Marsh, Yiyi Ma, Hans-Ulrich Klein, Sarah M. Connor, Alexandra Kroshilina, Christina J. Yung, Anthony Khairallah, Marta Olah, Julie Schneider, Kyle Karhohs, Anne E. Carpenter, Richard Ransohoff, David A. Bennett, Andrea Crotti, Elizabeth M. Bradshaw, Philip L. De Jager

**Affiliations:** 1 Center for Translational & Computational Neuroimmunology, Department of Neurology and the Taub Institute for Research on Alzheimer’s Disease and the Aging Brain, Columbia University Irving Medical Center, 630 West 168th st, PH19-311, New York, NY 10032 USA; 2 Cell Circuits Program, Broad Institute, Cambridge, MA USA; 3 Pacific Northwest National Laboratory, Richland, WA USA; 4 Biogen, 225 Binney St, Cambridge, MA 02142 USA; 5 Alzheimer’s Disease Center, Rush University Medical Center, Chicago, IL USA; 6 Imaging Platform, Broad Institute, Cambridge, MA USA; 7 Third Rock Ventures, 29 Newbury Street, Suite 301, Boston, MA 02116 USA; 8 Department of Cell Biology, Harvard Medical School, Boston, MA USA

**Keywords:** BIN1 isoforms, Alzheimer’s disease, Amyloid, Tau, Microglia, Astrocytes, Neurons

## Abstract

**Background:**

Identified as an Alzheimer’s disease (AD) susceptibility gene by genome wide-association studies, *BIN1* has 10 isoforms that are expressed in the Central Nervous System (CNS). The distribution of these isoforms in different cell types, as well as their role in AD pathology still remains unclear.

**Methods:**

Utilizing antibodies targeting specific BIN1 epitopes in human *post-mortem* tissue and analyzing mRNA expression data from purified microglia, we identified three isoforms expressed in neurons and astrocytes (isoforms 1, 2 and 3) and four isoforms expressed in microglia (isoforms 6, 9, 10 and 12). The abundance of selected peptides, which correspond to groups of BIN1 protein isoforms, was measured in dorsolateral prefrontal cortex, and their relation to neuropathological features of AD was assessed.

**Results:**

Peptides contained in exon 7 of BIN1’s N-BAR domain were found to be significantly associated with AD-related traits and, particularly, tau tangles. Decreased expression of BIN1 isoforms containing exon 7 is associated with greater accumulation of tangles and subsequent cognitive decline, with astrocytic rather than neuronal BIN1 being the more likely culprit. These effects are independent of the BIN1 AD risk variant.

**Conclusions:**

Exploring the molecular mechanisms of specific BIN1 isoforms expressed by astrocytes may open new avenues for modulating the accumulation of Tau pathology in AD.

## Introduction

**Fig. 1:**
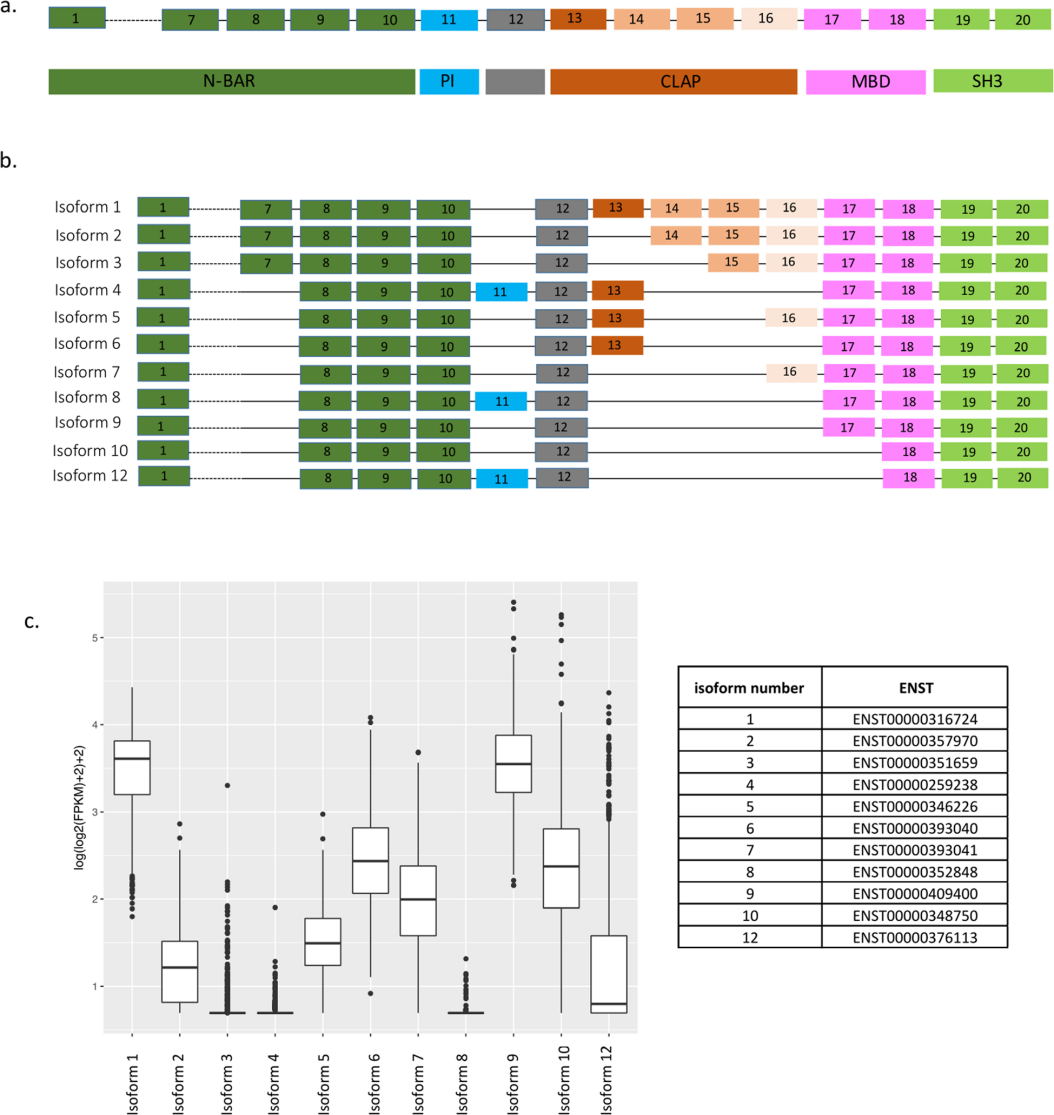
*BIN1* Isoforms. **a** The upper aspect of the panel shows the exonic structure of *BIN1* along the chromosome, with each exon numbered. Given space constraints, we do not show exons 2–6. The lower aspect of the panel highlights the different domains of the BIN1 protein. Both aspects of the panel are colored based on the functional domains. Glossary: N-BAR domain, phosphoinositide binding module (PI), clathrin and AP2 binding domain (CLAP), Myc-binding domain (MBD), and src homology 3 domain (SH3). **b** Diagrams of the 12 *BIN1* RNA isoforms. **c** mRNA expression of BIN1 isoforms in human dorsolateral prefrontal cortex (DLPFC) (*n* = 508 subjects) [6]. These are FPKM values from RNA-seq data, unadjusted for covariates (see Methods section); the data were obtained from subjects with pathological AD (58%) and subjects without AD pathology (42%), and the average age death of 88. We also provide a small table with the ENST reference number for each of the RNA isoforms considered in this study

Alzheimer’s disease (AD) is the most common form of aging-related dementia and is characterized by cognitive decline associated with hyperphosphorylation of Tau protein, accumulation of amyloid plaques and neurofibrillary tangles, and neuronal loss [1]. In the last few years, several susceptibility loci that contribute to genetic risk for AD, including one that contains the *BIN1* (Bridging integrator 1) gene, have been identified by genome wide association studies (GWAS) [2]. The effect size of the *BIN1* variant (tagged by rs6733839) is among the largest for common AD variants [3]; only *APOEε4* and *TREM2* [2] have larger effects. BIN1, a member of the BIN1/amphiphysin/RVS167 family, is highly expressed in the brain and in skeletal muscle [4]. It has been implicated in diverse cellular processes such as endocytosis, actin dynamics, DNA repair, membrane trafficking, inflammation and apoptosis [4, 5]. The *BIN1* gene has 20 exons which encode several known structures including an N-BAR domain, a phosphoinositide (PI) binding motif, a CLAP (clathrin and AP2) binding domain, a Myc-binding domain (MBD) and a Src homology 3 (SH3) domain (Fig. 1a). The N-BAR domain, encoded by exons 1 to 10, is involved in membrane curvature [7]. The phosphoinositide (PI) binding motif is encoded by exon 11 and is only present in a few BIN1 isoforms (isoform 4, 8 and 12). The CLAP domain is encoded by exons 13 to 16 and is involved in endocytosis [8]. Its inclusion in mature protein is highly variable among isoforms. Finally, the MBD domain, encoded by exons 17 and 18, plays a role in the regulation of c-Myc, a transcription factor regulating histone acetylation [9]. *BIN1* is expressed in 14 RNA transcripts, generated by alternative splicing, and 11 of them are translated into respective proteins: isoforms 1, 2, 3, 4, 5, 6, 7, 8, 9, 10 and 12 (Fig. 1b). Isoforms 1 through 7 are brain specific with isoform 1 being exclusively expressed in neurons [10]. Isoform 8 is specifically expressed in muscle [10]. Isoforms 9 and 10 are ubiquitously expressed and are also detected in the brain [11]. It was reported that BIN1 isoforms are translated into proteins differently in neurons and astrocytes: cultured neurons mostly express isoforms 1, 3, 5 and 7 while cultured astrocytes express isoforms 2, 5, 9 and 10 [11]. In addition, high expression of BIN1 has also been reported in mature oligodendrocytes, especially in the white matter of human brain [12].

Genetic analysis of *BIN1* has shown that the rs59335482 insertion/deletion variant is associated with an increase of *BIN1* mRNA expression in the brain [13], and this manuscript also reports an increase of *BIN1* mRNA expression in the central nervous system (CNS) of AD patients compared to non-AD patients [13]. These findings highlight that a polymorphism associated with an increase of BIN1 expression in the CNS is associated with AD risk. A different study found altered methylation in the *BIN1* locus at cg22883290 in relation to neuritic amyloid plaques and a pathologic diagnosis of AD, and this altered methylation pattern was independent of the effect of the rs744373 susceptibility variant [14]. Thus, there appears to be a convergence of a genetic factor and an environmental or experiential factor mediated by epigenomic changes on risk of AD in the *BIN1* locus. Further, mechanistic dissection has shown that BIN1 interacts with Tau [15] and is involved in Tau-mediated neurotoxicity in a *Drosophila melanogaster* model [13].

In addition to *BIN1*, GWAS have also identified over 30 other loci implicated in AD [3], and approximately one-third of them are expressed in microglia and other myeloid cells [3, 16, 17], emphasizing the potential role of immune cells as contributors to the onset of AD. BIN1 has been implicated in inflammation: there is a report of an increase of inflammation in *BIN1* knock-out mice [18]. However, the role of BIN1 in microglia needs to be clarified as at least some of its isoforms are expressed in all CNS cell types. In addition, an association between expression of some *BIN1* RNA isoforms and AD was reported: a decrease of isoform 1 and an increase of isoform 9 expression was noted in small collections of human AD *post-mortem* brain samples [19, 20]. De Rossi and colleagues reported that the decrease of isoform 1 (the largest isoform) protein expression is correlated with a decreased expression of a neuronal marker, illustrating the loss of neurons observed in AD. On the other hand, the level of isoform 9 seems to be correlated with the abundance of astrocyte (GFAP) and microglia (CD45) markers [12]. These findings suggest that there may be an increase of isoform 9 expression by reactive astrocytes and microglia at the protein level during AD. Indeed, *BIN1* mRNA encoding isoform 9 is highly transcribed by glial cells [21, 22]. BIN1 expression has been reported in IBA1+ cells in AD brain [13]. These cells may be microglia, but, upon review of the published images, these IBA1+ cells may be more representative of perivascular macrophages than microglia based on morphology [13]. Nonetheless, BIN1 is clearly expressed in myeloid cells of the CNS. Finally, the extent of the characterization of the expression of these different BIN1 isoforms in different cell types has been limited by the small number of commercially available antibodies against exon-selective epitopes of BIN1. Here, using antibodies recognizing specific exon-selective epitopes of BIN1 and a targeted proteomic approach, we studied the distribution of different translated BIN1 exons and hence the protein isoforms in different cell types in the human brain as well as their relation to pathologic measures of AD.

## Material and methods

### BIN1 isoform cloning

pLVX Ires-Neo lentiviral plasmid from Clontech was cut with EcoRI/MluI to remove the IRES-neo cassette. eGFP-P2A linker expression cassette with a new multiple cloning site was synthesized as linear dsDNA (gBLOCK (IDT)) and cloned into the cut vector using NEB Builder DNA assembly master mix (New England Biolabs). All BIN1 isoforms were then synthesized as gBLOCKs (IDT) and cloned into the GFP-P2A lentiviral vector using NEBuilder DNA assembly master mix according to the manufacturer’s protocol.

### Generation of BIN1 antibodies

Anti-BIN1 polyclonal Antibodies were generated by New England Peptides (Gardner, MA). Antigens were designed in-silico to produce the highest level of immunogenicity against each exon of interest. Synthesized ~ 15-mer peptides representing each exon of interest were injected in New Zealand White – SPF Rabbits (three immunizations of two rabbits, for each exon). Two production bleeds (40–50 mL antiserum) per rabbit were obtained and anti-BIN1 polyclonal antibodies were affinity-purified using~ 15-mer peptides specific for each exon.

### Cell transfection and Western blot

HEK 293 T cells plated in 10-cm dishes were transfected with 12 μg of lentiviral vector expressing various BIN1 isoforms using 36 μL Lipofectamine 2000 (Life Technologies). After 48 h, cells were harvested, and protein lysates were prepared from 1 × 10^6^ 293 T cells by adding 100 μL of Lysis Buffer (NaCl 140 mM, HEPES 10 mM pH 7.4, NP-40 1%) supplemented with PMSF 1 mM, DTT 1 mM, Complete Protease Inhibitor Cocktail (Roche). After a 1 h incubation on ice, lysates were cleared by spinning at 13,000 rpm, 10 min, 4 °C. Protein concentrations had been determined using a BCA protein assay (Biorad). A total of 20 μg of protein was separated by SDS-polyacrylamide electrophoresis using NuPAGE 7% Tris-Acetate Gel (Thermo-Fisher), then transferred to a Nitrocellulose membrane. After 1 h of blocking in 5% (w/v) non-fat milk in TBS with 0.1% Tween 20, the membranes were incubated overnight at 4 °C with in-house generated primary antibodies against various epitopes of BIN1 at a final concentration of 2 μg/ml or an anti-BIN1 antibody at a 1:500 dilution (99D and H100, Santa Cruz), respectively. The membranes were washed in TBST (4 × 10 min) before a 1 h incubation with an anti-rabbit HRP 1:5000 (Santa Cruz). The immunoblots were then visualized using the ECL (Amersham) (Suppl.Fig. 1a and b).

### Cell culture and TMA preparation

Preparation of cultured cells for paraffin embedding: a batch of 10 [9] 293Expi cells had been transfected with lentiviral vectors expressing various BIN1 isoform according to manufacturer’s instructions (Thermo-Fisher). After 48 h in culture, cells had been harvested, centrifuged at 1000 rpm at 4 °C for 5 min and washed in PBS twice. Once PBS had been removed, 10% Neutral Buffered Formalin was added down the side of the tubes onto the cell pellets (Thermo-Fisher). Formalin-Fixed cell pellets were Paraffin-Embedded (FFPE), sectioned and Tissue microarray (TMA) blocks were included in triplicate (~ 2.25 mm core diameter). After a 24 h fixation period, cells were processed and embedded into paraffin. A tissue microarray (TMA) was constructed from formalin-fixed, paraffin-embedded cell pellet preparations. Triplicate 2.25 mm cores from each of the cell pellet blocks were punched and placed into the TMA block. Untransfected HEK 293 T cell pellet preparation was also included in triplicate. A non-related tissue sample was included in the TMA as an orientation marker.

### Validation of isoform-specific anti-BIN1 antibodies

**Fig. 2:**
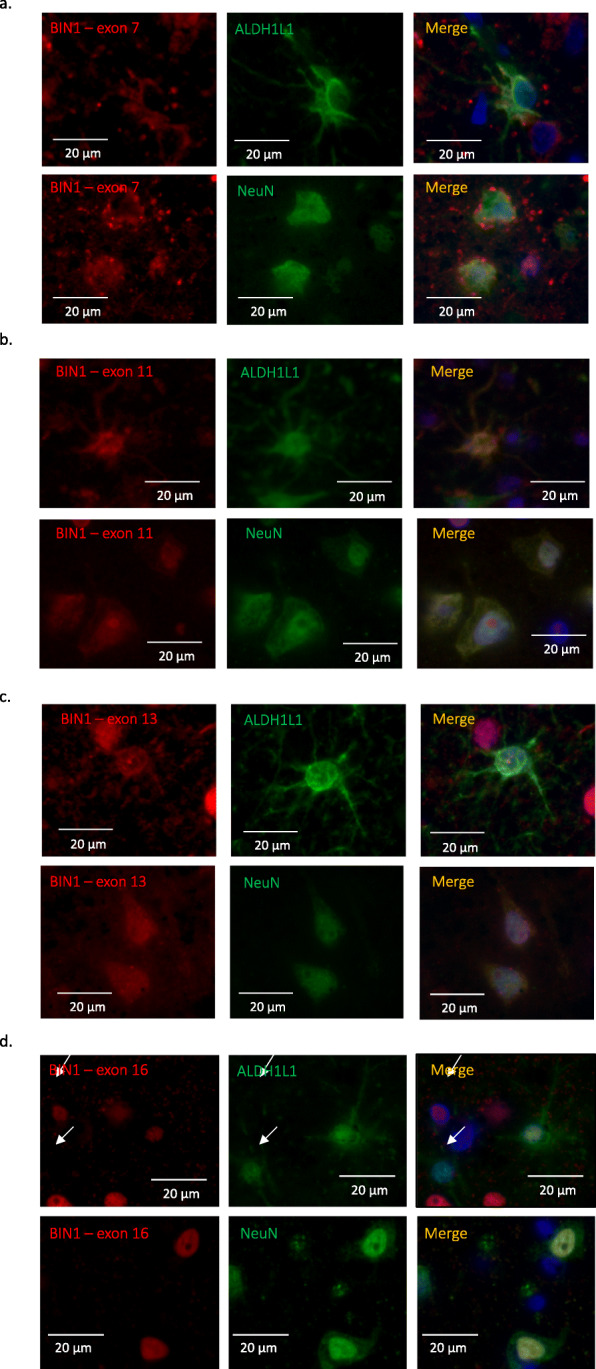
Characterization of the expression of BIN1 exons at the protein level in different brain cells. **a-d** Co-immunostaining using antibodies recognizing a specific exon of BIN1 (exons 7, 11, 13 or 16) in red with neuronal (NeuN) and astrocyte (ALDH1L1) markers in green in human *post-mortem* brain tissue. Cells which express both sets of markers have a yellowish color. Scale bar: 20 μm

Five-micron thick sections of TMA blocks containing 11 cell pellets were prepared and placed on charged slides. Immunohistochemical staining was performed using the Ventana Discovery Ultra automated staining platform (Roche Ventana Medical Systems, Tucson, AZ). Briefly, paraffin sections were deparaffinized and rehydrated. Epitope retrieval was performed in Ventana CC1 (EDTA, pH 8.0) buffer at 95 °C for 64 min. Slides were reacted against anti-BIN1 antibodies at one final concentration of 0.25–1 μg/mL, as selected from immunohistochemistry method optimization studies. Following primary antibody incubation, sections were incubated with secondary goat anti-rabbit antibodies (OmniMap HRP kit, Roche Ventana) followed by detection with diaminobenzidine (DAB) and counterstaining with hematoxylin. Stained slides were digitized, and staining was evaluated visually. Photomicrographs were captured at 100x magnification using ImageScope software (Leica Microsystems). A non-related tissue sample was included as an orientation marker (Suppl.Fig. 2).

### ROSMAP cohort

The Religious Orders Study (ROS) and Memory and Aging Project (MAP) are both cohort studies directed by Dr. David Bennett at RUSH University. Recruited non-demented individuals over the age of 65 consented to brain donation at the time of death. Both cohorts implement the same annual evaluations including 19 cognitive functional tests and use accepted and validated procedures to diagnose AD and other dementias and mild cognitive impairment (MCI). Since 1994 (ROS) and 1997 (MAP), more than 3069 individuals have been enrolled and 1641 are still alive. The follow-up rate exceeds 95% and the autopsy rate exceeds 90%. All participants undergo a uniform structured evaluation for AD including CERAD, Braak Stage, NIA-Reagan, and a global measure of AD pathologic burden with modified Bielschowsky; amyloid load and PHF Tau tangles by immunocytochemistry and image analysis and stereology; Lewy bodies and TDP-43 by immunocytochemistry; and the age, location, and size of all macro- and microscopic infarcts. Both frozen and fixed brain tissue is available upon request from these subjects. Our laboratory has generated DNA methylation scans, H3K9Ac ChipSeq, and RNAseq data (*n* = 508) from the frozen dorsolateral prefrontal cortex as part of other studies (Suppl.Table.1.a). We also have RNA-seq data from FACS-purified viable microglia from some of these individuals at autopsy (*n* = 21) (Suppl.Table.1.b).

### Immunohistochemistry on human *post-mortem* brain tissue

Formalin-fixed *post-mortem* brain tissues were obtained from brains donated to New York Brain Bank at Columbia University. All participants consented to brain donation at the time of death. The average age of death for all subjects in the study was 70 years old, with AD subjects meeting the criteria for pathologic AD by the NIA Reagan criteria (score 1–2). Cognitively unimpaired subjects without AD pathology were selected as control subjects (NIA Reagan score 3–4) (Suppl.Table.1.b).

Six μm sections of formalin-fixed paraffin-embedded tissue from the frontal cortex (*n* = 3) were used to stain for NeuN (Millipore), ALDH1L1 (eBioscience), CD45 (Novus Biological), along with anti-BIN1 antibodies provided by Biogen.

Immunohistochemistry was performed using citrate for antigen retrieval. The sections were blocked with blocking medium (8% of horse serum and 3% of BSA) and incubated overnight at 4 °C with primary antibodies. Sections were washed with PBS and incubated with fluorochrome conjugated secondary antibodies (Thermo Fisher) and coverslipped with anti-fading reagent with DAPI (P36931, Life technology). Photomicrographs are captured at 20x magnification using Zeiss Axio Observer Z1 fluorescence microscope and exported to Image J imaging software [23] (NIH, Maryland, USA). The images were quantified using CellProfiler and CellProfiler Analyst [24].

### Isolation of human monocytes and MDMi cell differentiation

Peripheral blood mononuclear cells (PBMC) were extracted from blood samples from the PhenoGenetic cohort using a standard Ficoll protocol (Ficoll-PaqueTM PLUS). PBMCs were frozen at a concentration of 1-3 × 10^7^ cells/mL in 10% DMSO + 90% FBS and stored in − 80 °C until further processing. CD14+ monocytes were purified from frozen PBMC samples using a positive isolation strategy (CD14+ selection kit, Miltenyi). One hundred thousand monocytes were allocated per well in 96-well plates. To differentiate to MDMi (Monocyte Derived Microglia-like), monocytes were cultured in RPMI-1640 media supplemented with 10 ng/ml M-CSF, GM-CSF and NGF-β, as well as 100 ng/ml CCL2 and IL-34 for 10 days.

### Expression analysis by real-time RT-PCR

Total RNA was isolated using the RNeasy Plus Micro Kit (Qiagen, Germany), according to the manufacturer’s protocol. The expression of genes of interest was measured by qRT-PCR in duplicate. B2M was used as an internal control. 5 μL of the cDNA was amplified using 10 μL of TaqMan Fast Advanced Master Mix, 1.5 μL of primers and 4 μL of RNase free water. qRT-PCR was run on the Quantstudio 3 detection system (Applied Biosystems, USA).

### RNA-sequencing

Five hundred eight samples from human DLPFC obtained from Rush University were submitted to the Broad Institute’s Genomic Platform for transcriptome library construction following the dUTP protocol and Illumina sequencing, 5 μg of total RNA at 50 ng/uL with RNA Integrity Number (RIN) score of 5 or higher were submitted for cDNA library construction. Tophat has been used to aligne the whole genome reference (hg19) with an average sequencing depth of 50 million paired reads per sample. A number of Picard metrics have been collected from the alignment results to implement a parallel and automatic RNA-seq pipeline to obtain a higher quality of alignment and better estimation on gene expression levels. The gene and transcript expression levels were estimated by RSEM package using the Gencode V14 for the quantification process. The estimation of abundance is in FPKM (Fragments Per Kilobase of transcript per Million mapped reads) format.

### Isolation of adult human microglia, RNA-sequencing and data processing

Microglia were isolated from autopsy brain material as described by Olah et al., in Nature Communication, 2018. Fresh brain samples of AD (*n* = 18) and non-AD (*n* = 3) subjects meeting the criteria for pathologic AD by NIA Reagan criteria (AD: score 1–2; non AD: score 3–4) with an average age of death of 93 were obtained from Rush University (ROSMAP cohort) (Suppl.Table.1.b). Briefly, upon arrival of the autopsy brain sample, the cerebral cortex and the underlying white matter were dissected under a stereomicroscope. All procedures were performed on ice. Only microglia isolated from the grey matter of the dorsolateral prefrontal cortex (DLPFC) were used in this study (*n* = 21). The dissected tissue was first mechanically dissociated. Subsequently, myelin was depleted using anti-myelin magnetic beads, following which the cell suspension was enriched in microglia with anti-CD11b magnetic beads. Microglia were then sorted based on their characteristic CD11b and CD45 expression and a viability dye (7AAD) on a BD FACS Aria II sorter. Cells were sorted into a 96-well PCR plate containing lysis buffer. Following FACS the lysate was snap frozen on dry ice and stored at − 80 °C until further processing. Library construction was performed using the SmartSeq-2 protocol described in detail elsewhere (Olah et al.). Samples were sequenced on an Illumina HiSeq2500 platform with a read length of 100 bp and paired end reads. RNA-Seq reads in FASTQ format were inspected using FASTQC program. Barcode and adapter contamination, low quality regions (10 bp at beginning of each FASTQ read) were trimmed using the FASTX-toolkit. The STAR (v2.5.3a) aligner software was used to map paired-end reads to the reference genome (assembly GRCh38) using Ensembl gene annotation system (release 91). Transcription levels were estimated using the RSEM (v1.2.31) software and reported in Transcripts Per Million (TPM). Four out of 25 samples were excluded due to a low percentage of exonic and UTR reads (< 30%) or a low total number of reads mapping to the transcriptome (< 106 reads).

### LC-MS/MS shotgun proteomics in purified human microglia

Label-free LC-MS/MS shotgun proteomics was performed using purified microglia isolated from fresh brains of AD (*n* = 1, NIA Reagan scale = 2) and non-AD (*n* = 1, NIA Reagan scale = 3) subjects based on NIA Reagan criteria with an average age death of 70. 5000 CD11b+/CD45+/7AAD- cells were sorted and lyzated and extracted by adding 10 μL of homogenization buffer (8 M urea, 10 mM dithiothreitol, 50 mM Tris pH = 8). After denaturation, protein solution was quantitatively transferred to a low retention LC vial (Waters) using 20 μL of 50 mM Tris pH = 8. Then, 25 μL of sample was added onto a 150 μm × 2 cm immobilized enzyme reactor for digestion followed by a separation of peptides using a 180 min gradient on an in-house 50 μm × 75 cm C18 (Phenomenex) analytical column. The SNaPP system was coupled to an Orbitrap Fusion Lumos mass spectrometer (Thermo Scientific). Fragmentation was carried out in the ion trap with maximum injection time of 250 ms for MS2 to increase sensitivity for low intensity ions. The peptides were identified using the MaxQuant software with a false discovery Rate (FDR) threshold of 1%.

### SRM proteomics in DLPFC

SRM proteomics was performed using frozen tissue from the DLPFC, *n* = 1377 (Suppl.Table.1). The sample preparation for LC-SRM analysis follows standard protocol, as described elsewhere [25, 26]. An average of ~ 20 mg of brain tissue from each subject was homogenized in a denaturation buffer (8 M urea, 50 mM Tris-HCl pH 7.5, 10 mM DTT, 1 mM EDTA). Following denaturation, 400 μg protein aliquots were taken for further alkylation with iodoacetamide and digestion with trypsin. The digests were cleaned using solid phase extraction, following readjustment of tryptic peptide digests concentration to 1 μg/μL. 30 μL aliquots were mixed with 30 μL synthetic peptide mix. All liquid handling steps were performed in 96-well plate format using Epmotion 5075 TMX (Eppendorf) and Liquidator96 (Rainin). We selected 7 proteotypic peptides based on BIN1 isoform-specificity and peptide detectability prediction tool CONSeQuence [27] (Suppl.Table.2). The 7 synthetic heavy peptides labeled with ^13^C/^15^N on C-terminal lysine and arginine were purchased from New England Peptide (Gardner, MA) with cysteine carbamidomethylation modification. All LC-SRM experiments were performed on a nano ACQUITY UPLC coupled to TSQ Vantage MS instrument, with 2 μL of sample injection for each measurement. A 0.1% FA in water and 0.1% in 90% ACN were used as buffer A and B, respectively. Peptide separations were performed by an ACQUITY UPLC BEH 1.7 μm C18 column (75 μm i.d. × 25 cm) at a flow rate 350 nL/min using gradient of 0.5% of buffer B in 0–14.5 min, 0.5–15% B in 14.5–15.0 min, 15–40% B in 15–30 min and 45–90% B in 30–32 min. The heated capillary temperature and spray voltage was set at 350 °C and 2.4 kV, respectively. Both the Q1 and Q3 were set as 0.7 FWHM. The scan width of 0.002 m/z and a dwell time of 10 ms were used. All the SRM data were analyzed by Skyline software [28]. All the data were manually inspected to ensure correct peak assignment and peak boundaries. The peak area ratios of endogenous light peptides and their heavy isotope-labeled internal standards (i.e., L/H peak area ratios) were then automatically calculated by the Skyline software and the best transition without matrix interference was used for accurate quantification. The peptide relative abundances were log2 transformed and centered at the median.

### Statistical analysis

Our statistical analyses were performed primarily using linear regression models that are adjusted for age, sex, cohort (ROS or MAP) as well as technical covariates that are specific to the data being analyzed. For RNA-seq data, we included RIN score, log2 (total aligned reads), and *post-mortem* interval. The table below describes the specific model deployed to test the relationship of a BIN1 measure with human traits in the analyses presented in the various figures and tables.

**Table:** 

Studies	Statistical Analysis Descriptions	Figures/Tables
**BIN1 isoform expression in DLPFC**	There are FPKM values from RNAseq processing pipeline, unadjusted for covariates. Briefly, RNA was captured with next generation RNA-seq on the illumina HiSeq platform. Samples with RNA integrity score < 5 or quantity threshhold < 5ug we excluded, Fragments per kilobase were corrected for any batch effect with Combat. These FPKM values were used for analysis here in models further adjusting for demographic and technical covariates. Further details on this pipeline can be found elsewhere: pubmed/24508835; pubmed/26414614.	Fig.1c
**Association of BIN1 isoforms with AD traits in DLPFC at RNA level**	Linear regressions of neuropathology and cognitive characteristics (cognitive decline, MMSE, neuritic plaque, amyloid burden, tau burden and neurofibrillary tangles) versus 11 expressed isoforms of BIN1 in DLPFC derived using RNA-seq data. Models were adjusted for age at death, sex, cohort (ROS/MAP), and technical RNA processing covariates.	Suppl. Fig. 4
**Measurement of BIN1 peptides abundance in DLPFC and its association with AD traits**	Linear regressions of BIN1 peptides versus neuropathologies and cognitive characteristics (clinical AD, cognitive decline, residual cognition (adjusting for pathology), pathological AD diagnosis post mortem, amyloid burder, and tau burden), adjusting for age at death, sex, and cohort (ROS or MAP).	Fig. 4, Suppl. Table. 5
**Association between BIN1 peptides and non-AD neuropathologic traits**	Linear regressions of BIN1 peptides versus other neuropathologies (cerebral amyloid angiopathy, macro infarct, micro infart, lewy bodies, hippocampal sclerosis, and TDP), adjusting for age at death, sex, and cohort (ROS or MAP).	Suppl. Table. 6
**Association of 7 BIN1 peptides with AD pathologies after adjusting for cell type proportion**	Linear regressions of BIN1 peptides versus neuropathologies and cognitive characteristics (clinical AD, cognitive decline, residual cognition (adjusting for pathology), pathological AD diagnosis post mortem, amyloid burder, and tau burden), adjusting for age at death, sex, and cohort (ROS or MAP), as well as myeloid proportion, neuron proportion, astrocyte proportion, oligodendrocyte proportion, and endothelial cell proportion as estimated using DSA with RNA-seq.	Suppl. Table. 7
**Association between BIN1 peptides encoded by exon 7 and tangles/amyloid after adjustment with tangle/amyloid burdens**	Linear regressions of peptides versus tau burden, adjusting for age of death, sex, and amyloid burden, as well as linear regressions of peptides versus amyloid burden, adjusting for age, sex, and tau burden.	Fig. 5a, Suppl. Table. 8
**Correlation between BIN1 exon 7/tangle burden/cognitive decline**	Linear regressions of BIN1 peptides versus cognitive decline (which is adjusted for age at death, sex, and education in a random effects model) first without adjusting for tau, and then adjusting for tau. R2s reported are partial R2s.	Suppl. Table. 9, Fig. 5 b,c
**Correlation between BIN1 exon 7/APOE4/cognitive decline**	Linear regressions of BIN1 peptides, and APOEe4 allele count, versus cognitive decline (which is adjusted for age at death, sex, and education in a random effects model), modeled independently, as well as combined in a joint model. R2s reported are partial R2s.	Fig. 5 d, Table. 1
**Study of the effect of AD susceptibility variants on BIN1 peptides in DLPFC**	Linear regressions of BIN1 peptides versus IGAP SNPs (Lambert et. *al.,* ) and Marioni family history of Alzheimer’s SNPs (Marioni et. *al.,* ), adjusted for principal components.	Suppl. Table. 10
**Evaluation of the effect of three BIN1 SNPs associated with AD on BIN1 isoform mRNA expression in DLPFC and in purified microglia**	Generalized linear regression was conducted to analyze the association between the genotype and expression level with the adjustment of the age at death, sex, post mortem interval, and major ethnicity principal components	Suppl. Fig. 3 b, c
**Evaluation of the effect of BIN1 SNPs associated with AD identified by GWAS on BIN1 isoform expression at the mRNA level in DLPFC**	The analysis included the filtered 898 single nucleotide polymorphisms (SNPs) (imputation quality ≥ 0.9, minor allele count ≥ 10, and minor allele frequency ≥ 0.05) within the genomic region of ±100 Kb region close to BIN1 (chr2: 127,705,602-127,964,931, Build hg19). The number of SNPs with Bonferroni corrected significance (p<0.05/898=5.57E-5) and nominal significance (p<0.05) were presented. The analysis was adjusted for age at death, sex, and major ethnic principal components.	Suppl. Table. 4 a, b
**Association of the BIN1 peptide LQAHLVAQTNLLR encoded by exon 7 with tangles**	Linear regressions of tau burden versus BIN1 peptides, adjusting for age at death, sex, as well as miRNA, and DLPFC modules, based on RNA-seq, which were previously shown to be associated with tau burden in this cohort (ROS/MAP).	Suppl. Table. 11
**Study of association between Tau-related epigenomic alterations and BIN1 peptides.**	Linear regressions of BIN1 peptides versus ETES (Epigenomic Tau Effect Score), adjusting for age at death, and sex.	Suppl. Table. 12

Table 1

**Table 1:** Relationship between BIN1 peptides and cognitive decline is not independent of *APOEε4*. The tables present regressions results of BIN1 peptides and count of APOEε4 alleles (0, 1, or 2) after adjustement for age at death, sex and cohort (ROS/MAP)

Effect of APOE휺4 on cognitive decline			
	**Beta**	***p***	**r2**
	-0.0446	3.37E-34	0.0571
Effect of BIN1 peptide on cognitive decline			
**peptide**	**Beta**	***p***	**r2**
LQAHLVAQTNLLR	0.0564	4.63E-05	0.0148
NQAEEELIK	0.0775	7.34E-06	0.0179
AAPQWCQGK	0.0287	0.013	0.00553
AEEELIK	-0.00473	0.134	0.00201
AQPSDNAPAK	-0.00497	0.254	0.00117
VNHEPEPAGGATPGATLPK	-0.00547	0.365	0.000736
GPPVPPPPK	0.0262	0.0605	0.00315
Effect of APOE휺4 on cognitive decline after adjustment with BIN1 peptide			
**peptide**	**Beta**	***p***	**r2**
LQAHLVAQTNLLR	-0.0508	2.23E-16	0.059710486
NQAEEELIK	-0.051	1.10E-16	0.06085979
AAPQWCQGK	-0.0519	2.65E-17	0.063426582
AEEELIK	-0.0526	1.39E-17	0.064351255
AQPSDNAPAK	-0.0526	1.78E-17	0.064331654
VNHEPEPAGGATPGATLPK	-0.0528	1.20E-17	0.064654118
GPPVPPPPK	-0.0532	5.21E-18	0.065998496
Effect of BIN1 peptide on cognitive decline after adjustment with APOE휺4			
**peptide**	**Beta**	***p***	**r2**
LQAHLVAQTNLLR	0.0391	0.00386	7.60E-03
NQAEEELIK	0.0595	0.000392	1.14E-02
AAPQWCQGK	0.0238	0.0325	4.18E-03
AEEELIK	-0.00275	0.366	7.46E-04
AQPSDNAPAK	-0.000636	0.88	2.08E-05
VNHEPEPAGGATPGATLPK	-0.00202	0.73	1.09E-04
GPPVPPPPK	0.0302	0.0247	4.59E-03

## Results

### Distribution of BIN1 isoforms in the CNS using transcriptomic and proteomic approaches

We first evaluated the distribution of BIN1 isoforms in the CNS by analyzing RNA expression of each isoform in human dorsolateral prefrontal cortex (DLPFC) (Fig. 1c); the figure also provides the reference numbers for each BIN1 isoform and an outline of the locus. Isoforms 1 and 9 are the ones most highly expressed, followed by isoforms 6, 7 and 10. Isoforms 2, 3, 5, and 12 are expressed at low levels, and isoform 4 and isoform 8, which has been reported to be specific to muscle [10], are absent.

To characterize the cell-specific expression of BIN1’s different isoforms at the protein level in human *post-mortem* tissue, 4 antibodies against 4 specific exons (exon 7, 11, 13 and 16) were generated. The targeted exons are present in different domains of BIN1: exon 7 is present in the N-BAR domain; exon 11 is in the PI domain; while exons 13 and 16 are in the CLAP domain (Suppl.Fig. 1a). These exons are alternatively spliced in selected isoforms (Fig. 1b). To validate the various BIN1 antibodies, we cloned BIN1’s 11 protein-coding isoforms and expressed them in HEK 293 T cells. As positive controls, we used commercially available anti-BIN1 antibodies: clone 99D (recognizing a mid-portion of BIN1 present in isoforms 1 through 9) and clone H100 (recognizing the C-terminus of BIN1 present in isoforms 1 through 12). No basal expression of BIN1 was detected in the HEK 293 T cell clone that we used to express the different isoforms. As expected, the 4 custom antibodies against different epitopes of BIN1 recognized the selected isoform, according to the scheme depicted in Fig. 1b (Suppl. Fig. 1b). Furthermore, the 4 custom antibodies against different epitopes of BIN1 recognized the selected isoform expressed in HEK 293 T cells by immunohistochemistry (Suppl. Fig. 2). Specific reactivity was demonstrated against the appropriate transfected cell pellet controls, while no non-specific reactivity was observed (Suppl. Fig. 2). Having validated these new reagents, we deployed them to stain human *post-mortem* brain samples to characterize the distribution of the different isoforms of BIN1 by immunofluorescence. We note that staining of oligodendrocytes could not be appreciated relative to background staining, and we therefore elected not to characterize BIN1 protein expression further in this cell type.

### Specific BIN1 isoforms are expressed in neurons and astrocytes

Immunostaining using the antibody recognizing exon 7 showed co-localization with NeuN, a neuronal marker, and with ALDH1L1, an astrocyte marker (Fig. 2a). This observation suggests that exon 7 of BIN1 is expressed at the protein level in both neurons and astrocytes. The same approach was used for the antibodies recognizing exons 11, 13 and 16 which are also expressed in both neurons and astrocytes (Fig. 2b, c, d). Considering that exon 7 is only present in BIN1 isoforms 1, 2, and 3 (Fig. 1b), the results indicate that these three isoforms might be expressed in neurons and astrocytes. BIN1 isoform 12, which contains exon 11 (Fig. 1b), is also expressed in neurons and astrocytes. Similarly, isoforms 5 and 6 that contain exon 13, and isoform 7 which contains exon 16 (Fig. 1b) appear to be expressed in both cell types. Since isoforms 4 and 8 are not expressed in the CNS (Fig. 1c), we excluded them from this annotation relating to neurons and astrocytes. In addition, we observed that antibodies recognizing different exons are detected in different subcellular compartments in astrocytes and neurons (nuclear for exons 13 & 16 and cytoplasmic for exons 7 & 11), confirming the observation reported by other studies that BIN1 is found both in the nucleus and cytosol [29–31].

### BIN1 isoforms 6, 9, 10 and 12 are expressed in human microglia

**Fig. 3:**
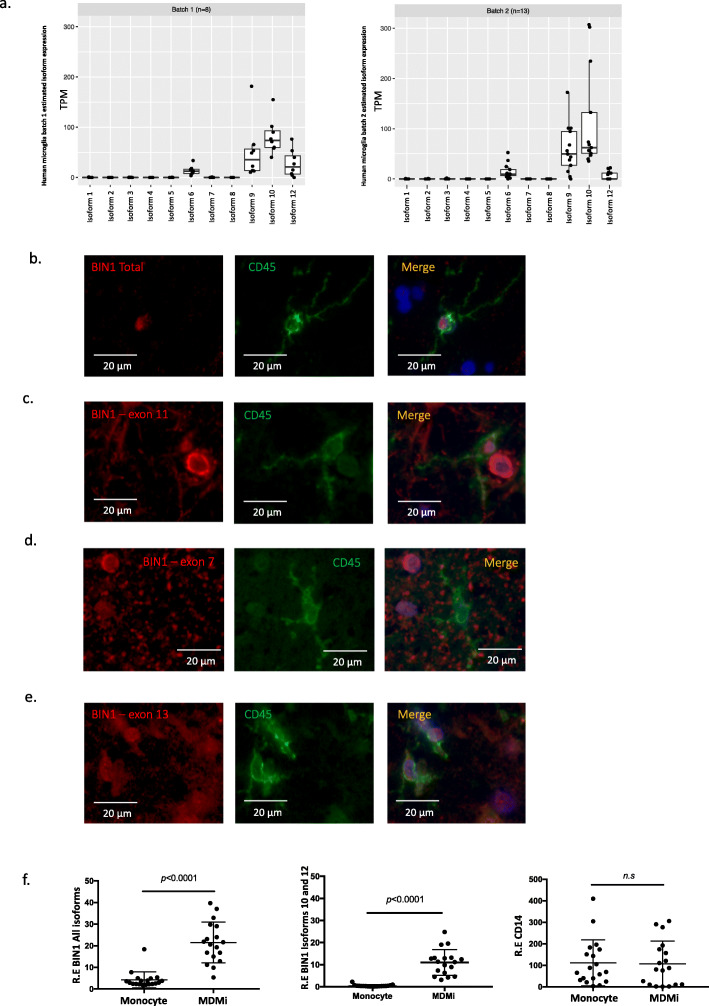
BIN1 expression in microglia. **a** RNA expression of BIN1 isoforms expressed in human primary microglia isolated from human fresh autopsy tissue. Two different batches of human microglia with *n* = 8 subjects on the left and *n* = 13 subjects on the right showed comparable results. **b-e** Co-immunostaining in *post-mortem* human brain tissue (*n* = 3) with antibodies recognizing specific BIN1 exons in red (exons 7, 11, 13 or 17, which recognizes isoforms 1–9) and the microglial marker CD45 in green. Cells which express both sets of markers have a yellowish color. **f** Analysis of gene expression level of BIN1 (all isoforms, including isoforms 10 and 12) in monocytes and MDMi (*n* = 19). No difference of gene expression level reported for CD14, a marker of myeloid cells, between two cellular models. Relative gene expression is reported on the Y-axis

In order to investigate whether BIN1 is expressed in microglia, we performed a BIN1 mRNA isoform expression analysis in transcriptomes generated from purified live grey matter microglia purified from fresh 21 autopsy samples [16] (see Suppl.Table.1.b. for description of these subjects). Using estimates of isoform abundance derived from RNA-seq data, we observed expression of isoforms 6, 9, 10 and 12 in these purified cortical microglia, with isoform 10 being the most highly expressed isoform in both batches of samples (Fig. 3a). Estimates of isoform expression level from these RNA-seq data are supported by the results of an alternative approach which counts the number of reads mapped to each exon (Suppl. Fig. 3a). Overall, these results support the presence of *BIN1* mRNA in purified human microglia, and shotgun proteomic data generated from purified microglia derived from other human samples by the same pipeline confirmed the detection of BIN1 at the protein level. As seen in Supplementary Table 3, 6 BIN1 peptides encoded by different exons were present in these proteomic data (see Suppl.Table.1.b for description of these subjects).

In order to explore the expression of BIN1 by microglia in human *post-mortem* brain tissue, an antibody recognizing an epitope of BIN1 contained within isoforms 1–9 (BIN1 Total) was used first in combination with the microglial marker CD45 (Fig. 3b). The results showed expression of BIN1 in the nucleus of CD45+ cells with a morphology consistent with microglia, suggesting the presence of BIN1 isoforms 1–9 in microglia. In order to narrow down the identity of the isoforms that are specifically expressed by microglia at the protein level, the exon specific antibodies of BIN1 were again utilized. The BIN1 antibody recognizing exon 11, contained in isoforms 4, 8 and 12 (Fig. 3c), produced a positive staining in the nucleus of CD45+ cells, confirming the expression of isoform 12 which was also observed at the mRNA level. Isoforms 4 and 8 also contain exon 11, but they can be excluded since we didn’t detect these isoforms at the RNA level in purified microglia (Fig. 3a). Subsequently, we tested a BIN1 antibody against exon 7 (which is present in isoforms 1, 2 and 3) but observed no co-localization between BIN1 and CD45 (Fig. 3d), suggesting that BIN1 isoforms 1, 2 and 3 are not expressed by microglia. These results confirm our analysis of microglia mRNA where none of these isoforms were detected (Fig. 3a) and are consistent with previously published reports of isoform 1 and 3 expression in neurons and isoform 2 in astrocytes [11]. Finally, we tested an antibody against exon 13 (present in isoforms 4, 5 and 6). As seen in Fig. 3e, we detected positive staining in the nucleus of CD45+ cells, suggesting that microglia express BIN1 containing exon 13 confirming our results of RNA expression analysis of microglia, where isoform 6 was detected. In summary, using antibodies against selected BIN1 epitopes, we find that BIN1 isoforms 6, and potentially 9 and 12 are expressed by microglia in the aged human brain.

To explore whether these isoforms are expressed in all myeloid cells (including microglia and monocytes) or whether they are more specific to myeloid cells of the CNS, we evaluated data from human purified primary monocytes and matched monocyte-derived microglia-like (MDMi) cells (Fig. 3f). We note a significant increase of BIN1 isoforms 10 and 12 in MDMi compared to corresponding monocytes. These findings showed that isoforms present in myeloid cells are expressed in a context-dependent manner and are probably more prominent in microglia than in peripheral myeloid cells. Table 2 presents a summary of these results.

**Table 2:** Summary table illustrating the distribution of BIN1 exons and isoforms in different cell types in CNS

	DLPFC - Bulk RNA-seq	Neuron – Immunofluorescence Exon Specific Antibody	Astrocyte – Immunofluorescence Exon Specific Antibody	Microglia - Purified Microglia RNA-seq	Microglia – Immunofluorescence Exon Specific Antibody
**Isoform 1**	Yes	exon 7	exon 7	No	No
**Isoform 2**	Yes	exon 7	exon 7	No	No
**Isoform 3**	Yes	exon 7	exon 7	No	No
**Isoform 4**	No	No	No	No	No
**Isoform 5**	Yes	exon 11, 13	exon 11, 13	No	No
**Isoform 6**	Yes	exon 11, 13	exon 11, 13	Yes	exon 11, 13
**Isoform 7**	Yes	exon 16	exon 16	No	No
**Isoform 8**	No	No	No	No	No
**Isoform 9**	Yes	data N/A	data N/A	Yes	data N/A
**Isoform 10**	Yes	data N/A	data N/A	Yes	data N/A
**Isoform 12**	Yes	data N/A	data N/A	Yes	data N/A

We evaluated the possible role of genetic variants on *BIN1* expression; in particular, we evaluated the BIN1 single nucleotide polymorphisms (SNPs) previously reported to be associated with AD. However, these variants had no effect on gene or isoform mRNA expression in a well-powered sample of cortical tissue data [6] (*n* = 508) or in the small sample of subjects with purified microglia RNA described above (*n* = 21) (Suppl. Fig. 3b and c). Other AD SNPs in the *BIN1* locus were seen to have a modest effect on expression in cortical tissue data for isoform 10, which is expressed in microglia (Suppl.Table.4a and 4b), but this was not seen in the small dataset derived from purified microglia.

### BIN1 isoforms 1, 2 and 3 are associated with tau tangles

Using RNA sequencing data from the DLPFC of 508 participants in two prospective studies of aging (ROSMAP subjects), we assessed the relation of BIN1 isoforms to AD-related traits (Suppl. Fig. 4). For most of the isoforms, we did not find significant association between *BIN1* isoforms and AD-related traits such as a person’s slope of cognitive decline prior to death, AD dementia or quantitative measures of tau tangle burden, which measures the presence of an AD-associated phosphorylated epitope of MAPT. Only BIN1 isoform 1 was significantly associated with residual cognition (a measure of how well someone is performing cognitively given their burden of aging-related neuropathologies [32]) and amyloid burden at the RNA level.

**Fig. 4:**
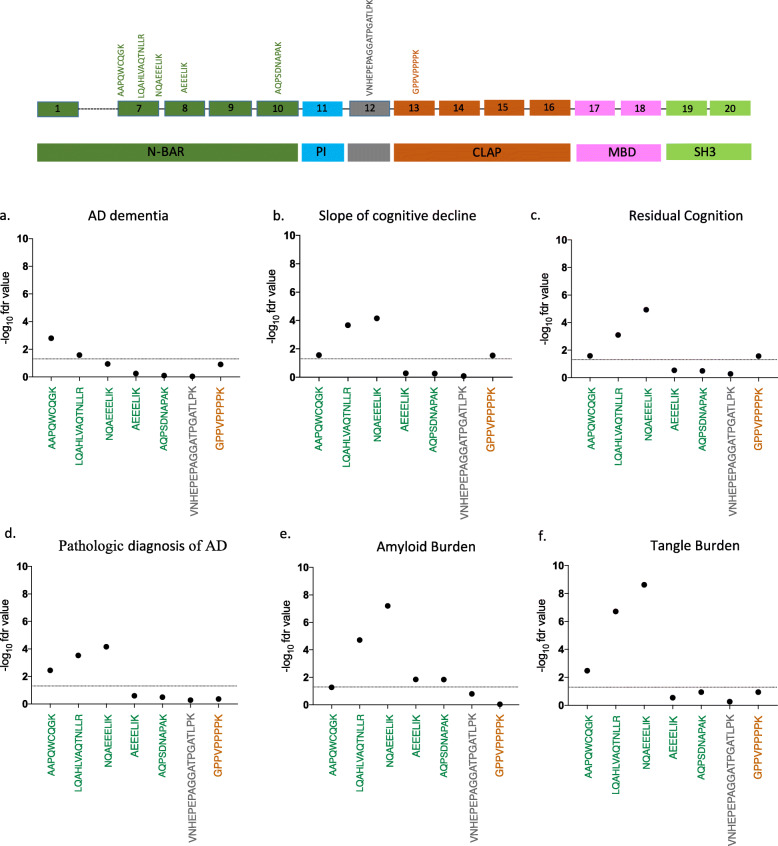
Quantitative analyses of BIN1 peptides in dorsolateral prefrontal cortex (DLPFC). The location and sequence of tested BIN1 peptides is shown in the diagram at the top of the figure. Peptides are colored based on the functional domains of the protein. **a-f** The abundance of each of the 7 peptides from 3 different domains of BIN1 was tested for association with different AD- related pathology traits in human DLPFC using SRM proteomics. The threshold of significance is reported on the Y-axis and is denoted by the horizontal dashed line as the -log of the False Discovery Rate (FDR) value (FDR < 0.05)

Given potential differences between RNA and protein expression, we then turned to a quantitative proteomic approach: we used signal reaction monitoring in DLPFC samples of a larger set of ROSMAP participants (*n* = 1377) to measure the abundance of 7 peptides that originate from different domains of the BIN1 protein (Fig. 4 and Suppl.Table.2–3). Five of the peptides map to the N-BAR domain: two in exon 7, one spanning exon 7 and 8 and one each in exons 8 and 10. The other two peptides map to a linker domain encoded by exon 12, as well as exon 13, which contains the proximal element of the CLAP domain.

After testing 7 peptides (Suppl. Fig. 5), only the three peptides which contain sequences from exon 7 display associations with AD related-traits: a diagnosis of AD dementia, the participants’ trajectory of cognitive decline, residual cognition and quantitative measures of both amyloid and tau pathology (Fig. 4a-f and Suppl.Table.5). In secondary analyses, we also evaluated other neuropathologic traits that are available in these subjects and find no association with neurovascular measures, cerebral amyloid angiopathy, hippocampal sclerosis, Lewy Bodies, or the burden of TDP43 pathology (Suppl.Table.6). These findings suggest a specific association between exon 7 of BIN1 and AD-related pathologies, and our measures of amyloid and tau tangles appear to be more strongly associated than the measures related to clinical function. Overall, as pathology burden increases, exon 7 peptide expression is reduced. Similarly, the expression of these peptides is diminished as cognitive functions worsens. To address the possibility that these associations are due to a shift in cortical cell populations, we repeated these analyses in a subset of individuals in which we could estimate the proportion of different cell types using RNA sequence data (*n* = 508). As seen in Supplementary Table 7, the results of these analyses remain significant for AD pathologies after including a correction for the different cell types.

**Fig. 5:**
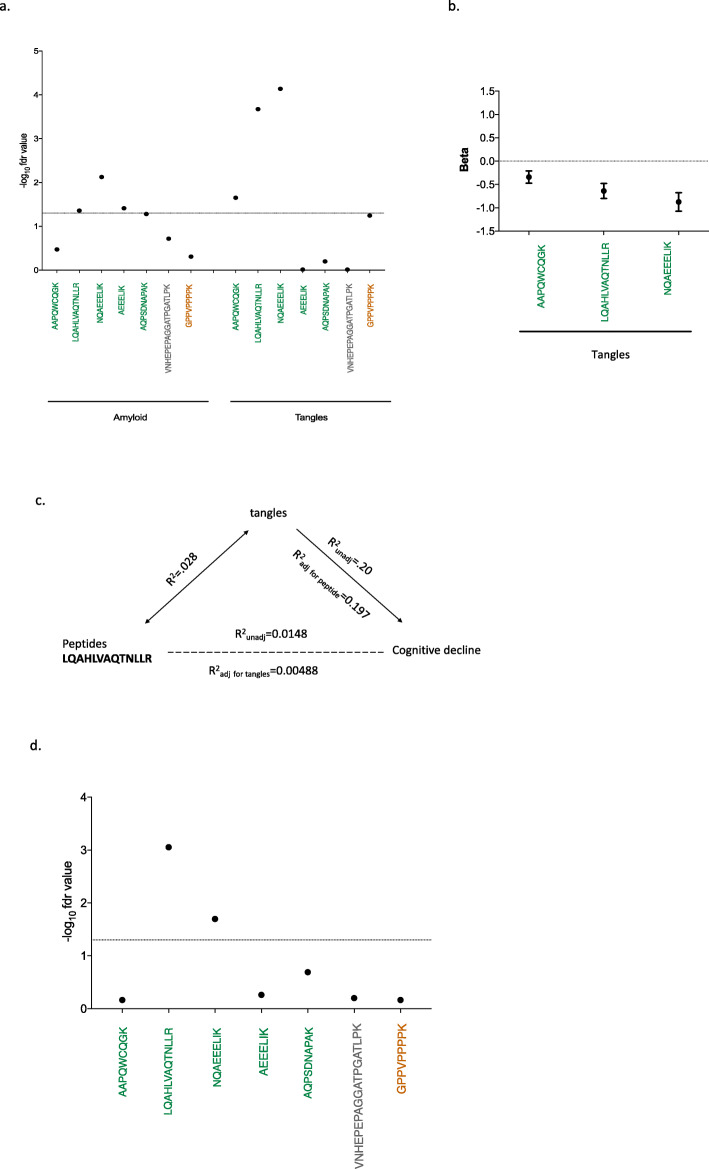
Association of BIN1 peptides with AD pathology. **a** Association between the abundance of BIN1 peptides and amyloid (left) or tangles (right) after adjusting for the other form of AD-related pathology. **b** Effect size of BIN1 peptides localized in exon 7 in relation to the abundance of tangles: there is an inverse relationship between the two factors. **c** Graph illustrating the results of our mediation analyses which suggest that BIN1 exon 7 peptides (i.e LQAHLVAQTNLLR) play a role in the accumulation of tangles and do not have an independent effect on cognitive decline. **d** Association of BIN1 peptides with *APOEε4.* The threshold of significance is reported on the Y axis and is denoted by the horizontal dashed line as the -log of the FDR value (FDR < 0.05)

Since the burden of amyloid and tau tangles are partially correlated to one another, we used conditional analyses to assess whether BIN1 exon 7 peptides are primarily driven by one of the two pathologies or are independently associated to both measures (Suppl.Table.8). When we adjust for the burden of tau tangles, most of the associations with amyloid are no longer significant, aside for the peptide that bridges exons 7 and 8, which is diminished by 49.2% (Fig. 5a). On the other hand, the associations with tangles are diminished but remain significant when we account for the effect of amyloid, suggesting that the association of the alternatively spliced exon 7 of BIN1 with AD is primarily driven by tangles, with a possible minor component involving amyloid independent of tangles (Fig. 5a).

Since the primary effect of BIN1 exon 7 on pathology may be exerted through tangles, we further explored whether this relation with tangles explained the association of BIN1 with cognitive decline. In evaluating different models using mediation analyses, we found that the peptide LQAHLVAQTNLLR explains 1.48% of the variance in cognitive decline (*p* = 4.63.10^− 5^) when modeled independently: with higher protein expression of this peptide we see slower cognitive decline. On the other hand, tangles explain 20% of the variance in cognitive decline (*p* = 7.59.10^− 67^), where higher tangles increase the rate of cognitive decline. When modeled jointly, the effect of the peptide LQAHLVAQTNLLR on cognitive decline is reduced significantly (from *r*^2^ = 1.48% to *r*^2^ = 0.49%), whereas the effect of tangles on cognitive decline is unchanged (from *r*^2^ = 20% to *r*^2^ = 19.7%), suggesting that the majority of the role of BIN1 in cognitive decline is not independent of tangles (Suppl.Table.9). In other words, we propose that reduced inclusion of exon 7 into BIN1 proteins contributes to increases in tangle burden (Fig. 5b), which then causes more rapid cognitive decline. However, we cannot exclude the possibility that the increase of tangle burden might lead to a decrease of exon 7 inclusion, which then causes cognitive decline.

Figure 5c presents a summary of these results and a proposed model which can help to craft hypotheses for future investigations.

### Effect of genetic variants and known risk-associated molecular features on peptide measures

We evaluated the role of *APOEε4* which has a strong role in AD, with both tangles and cognitive decline (Fig. 5.d): specifically, *APOEε4* explains approximately 7.7% of the variance in tangles while the BIN1 peptide LQAHLVAQTNLLR explains 2.8%. Similarly, LQAHLVAQTNLLR explains 1.48% of the variance in cognitive decline, while APOE*ε*4 explains 5.7% when they are assessed independently. In a conditional model containing both the LQAHLVAQTNLLR peptide and APOE*ε*4, the LQAHLVAQTNLLR peptide explains 0.76% of the variance in cognitive decline (Table 1), whereas APOE*ε*4 explains 6% of the variance in cognitive decline. This indicates that the association of LQAHLVAQTNLLR peptide with cognitive decline persists after adjustment with APOE*ε*4 (*p* = 0.00386) but is not entirely independent: APOE*ε*4 plays some role in the decrease of exon 7 inclusion in AD.

Secondarily, we evaluated whether the other known AD susceptibility variants influence BIN1 peptides, but we found no evidence of association between AD variants and BIN1 peptides after correcting for the testing of multiple hypotheses (Suppl.Table.10). Further, we performed a cis-pepQTL (+ − 1 Mb) (peptide QTL) analysis of the *BIN1* locus to find genetic variants that influence the abundance of exon 7, but we found minimal effects of genetic variation in the vicinity of *BIN1* on peptide expression. We found a single linkage disequilibrium (LD) block where the top SNP rs74490912 is associated with expression of BIN1 peptide LQAHLVAQTNLLR (*p* = 4.9 × 10^− 6^), but this SNP is only in weak linkage disequilibrium (LD) with the *BIN1* AD SNP rs6733839 (*R* [2] = 0.156). Thus, this genetic effect is not related to known AD genetic susceptibility.

In other analyses, we have found tau tangles to be associated with certain miRNA previously reported to be associated with AD (miR132 and mir129) [33], as well as certain modules of co-expressed genes derived from cortical RNA sequence data [6]. We therefore extended our analyses to include these variables, and we found that the association of the BIN1 peptide LQAHLVAQTNLLR with tangles is independent of all of these factors (Suppl.Table.11). Finally, we have recently reported large-scale changes in the neuronal epigenome in relation to tangles [34], and we have developed a person-specific score for the extent of cortical epigenomic alteration. Using this score, we found that there is no association between Tau-related epigenomic alterations (defined by the H3K9ac histone marks) and the LQAHLVAQTNLLR or other BIN1 peptides (Suppl.Table.12). Thus, the relation of BIN1 protein expression to the accumulation of tangles appears to be independent of these factors.

### Validating the association of exon 7 with AD in human *post-mortem* tissue

**Fig. 6:**
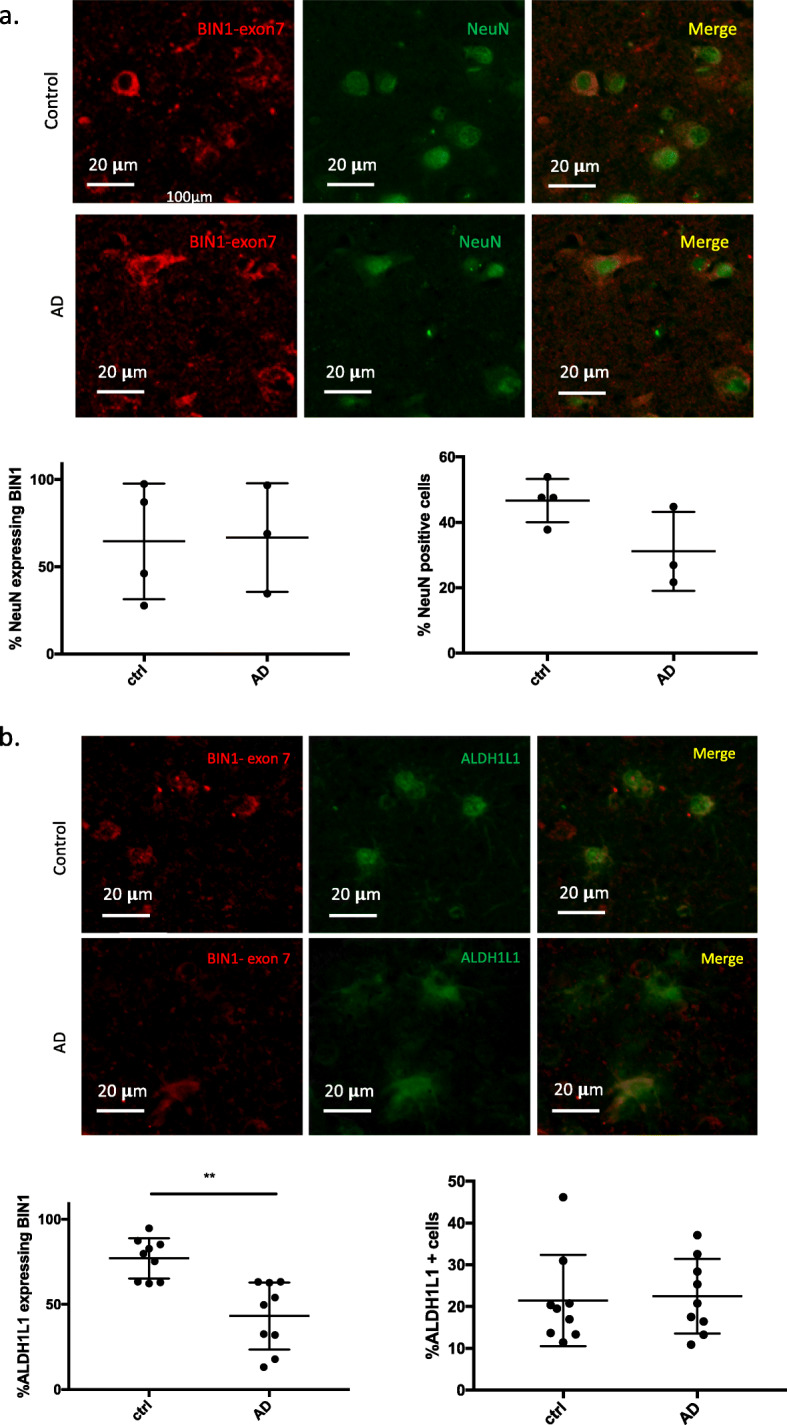
Association between BIN1 isoforms and AD pathology in brain *post-mortem* tissue. **a** Co-immunostaining using an antibody recognizing specifically BIN1 exon 7 in red and a neuronal marker (NeuN) in green in human *post-mortem* brain tissues for *n* = 3 AD and *n* = 4 non-AD subjects. The number of neurons expressing exon 7 and the total number of neurons have been quantified using the software CellProfiler and CellProfiler Analyst. **b** Co-immunostaining in human *post-mortem* brain tissues using an antibody recognizing specifically BIN1 exon 7 in red and an astrocyte marker (ALDH1L1) in green for *n* = 9 AD and *n* = 9 non-AD subjects. Cells which express both sets of markers have a yellowish color. The number of astrocytes expressing exon 7 and the total number of astrocytes have been quantified using the software CellProfiler and CellProfiler Analyst. A t-test has been used for statistical analysis using Prism software. The thresholds of significance are *p* < 0.05* and *p* < 0.005**

Given the association of exon 7 with AD and AD-related pathologies in the proteomic analyses, we stained AD and cognitively non-impaired subjects from the New York Brain Bank (see Suppl. Table.1.b for a description of these subjects) with the antibody recognizing BIN1 exon 7 and ALDH1L1, a marker of cortical astrocytes, or NeuN, a neuronal marker to resolve which cell type may be driving the association. We collected images in a systematic manner and used Cell Profiler, an image analysis software, to automatically segment these images and count the number of cells expressing each marker combination. We then compared the frequency of cells expressing each marker combination in the AD and cognitively non-impaired subjects. While the number of BIN1 exon 7+ neurons shows no difference between the two sets of subjects (with a total of 9735 neurons assessed in our 7 subjects) (Fig. 6a), we find, in the same individuals, a significant decrease in the number of astrocytes expressing BIN1 exon 7 in AD compared to cognitively non-impaired subjects (a total of 14,643 astrocytes were evaluated), while there is not a significant difference in the total number of astrocytes between the two classes of subjects (Fig. 6b). This suggests that the role of BIN1 exon 7 in AD is related to its role in astrocytes and perhaps that a loss of a class of BIN1 exon 7+ astrocytes contributes to the accumulation of tangle burden.

## Discussion

Since its identification as a genetic risk factor for AD in 2010*,* several studies have focused on characterizing the mechanism by which BIN1 contributes to the onset of AD [13, 19, 35]. Currently, only 2 of the 11 translated isoforms have been described as being differentially expressed in the AD brain: a decrease of isoform 1 and an increase of isoform 9 expression correlate with AD [19]. However, it remains unclear (1) whether these transcriptional changes are due to differences in cell type proportions and (2) in which cell type(s) BIN1 may play a role in influencing AD risk since it is widely expressed.

In this study, we identified the cell types expressing different BIN1 RNA and protein isoforms and investigated their association with AD neuropathology. At the RNA level, we detected expression of isoforms 1, 2, 3, 4, 5, 6, 7, 9, 10 and 12 in the human DLPFC, and we observed that isoforms 1 and 9 are highly expressed compared to the other isoforms. These observations independently validate what has been recently published [36]. Utilizing an antibody specifically targeting exon 7 on *post-mortem* human brain, we deduced that isoforms 1, 2 and 3 are expressed in both neurons and astrocytes. We cannot exclude the possibility that our antibody did not detect certain epitopes present at very low expression levels, such as isoforms 1, 2 and 3 in microglia; however, these isoforms are absent in microglia in our RNA-seq data from purified human microglia.

Exon 7 encodes a portion of the N-BAR domain of BIN1 and is specific to isoforms 1, 2 and 3 [7, 37]. Exon 7 enables the interaction between BIN1 and dynamin 2 (DNM2), a ubiquitously expressed GTP binding protein, and this interaction facilitates endocytic uptake [38]. Absence of exon 7 leads to the inhibition of uptake, suggesting that the BIN1 N-BAR domain plays a key role in endocytosis via its interaction with DNM2 [38]. In addition, a decrease of DNM2 mRNA in AD temporal cortex has been reported [39]. On the other hand, the BIN1 N-BAR domain has also been implicated in stabilizing the polymerization of actin, including the one induced by Tau [40]. Furthermore, downregulation of BIN1 in a Drosophila model reduced Tau accumulation [13]. This study connects BIN1 to Tau pathology, although the direction of effect is reversed in this Drosophila model system relative to what others and we have described in human samples. In our study, we have shown that the expression level of exon 7 peptides (AAPQWCQGK, LQAHLVAQTNLLR, NQAEEELIK) is associated with tangles and cognitive decline. In addition, we report that exon 7 peptides of BIN1 are negatively correlated with tangles, suggesting that decreased expression of neuronal/astrocyte isoforms 1, 2, and 3 contributes to greater accumulation of tangles and cognitive decline. Interestingly, BIN1 has been reported to regulate endocytosis in neurons, and the loss of its function appears to promote the propagation of Tau pathology in an in vitro model, supporting our findings [41]. However, our in situ validation revealed a loss of BIN1 exon 7 expression in astrocytes in AD subjects suggesting that the lack of expression of these specific BIN1 isoforms in astrocytes might be involved in AD pathology. Nonetheless, further studies should be performed to validate this finding by increasing the number of subjects, especially for BIN1 exon 7 staining in neurons in AD brains, and further investigation of the potential role of BIN1 isoforms expressed by astrocytes using in vitro and animal models is warranted.

While this study focused the expression of BIN1 in three major cell types in the grey matter of DLPFC (10.1101/566307), the expression of BIN1 by mature oligodendrocytes and its involvement in oligodendrocyte myelination has been described in the white matter [12]. These investigators reported that oligodendrocytes, which are enriched in white matter, mainly express BIN1 isoform 9 [42], which could explain why our exon-specific antibodies did not detect BIN1 isoforms expressed by oligodendrocytes, since exons 7, 11, 13 or 16 are not contained within isoform 9.

In summary, based on our data and evidence from the literature, we propose that decreased expression of isoforms 1, 2, and 3 leads to an increase in the endocytosis process and promotes the phosphorylation of AD-related Tau epitopes in neurons, either in a cell autonomous or non-cell autonomous fashion. The potential function and mechanisms of these BIN1 isoforms expressed in astrocytes should be investigated further. Overall, our model implicates BIN1 in the narrative of altered endocytosis that is suggested by several of the known AD susceptibility loci. However, the BIN1 AD variant itself does not appear to function through these alterations in the splicing of the N-BAR domain: the rs6733839 variant’s mechanism remains unclear and independent of the role of the N-BAR domain in AD.

Given our results, BIN1’s expression in microglia may be unrelated to the convergence of other AD risk factors in microglia, although we need larger microglia-specific datasets to fully explore the function of microglial BIN1 in AD. Nonetheless, we have enriched our understanding of BIN1 in this study: our multidisciplinary approach identified 4 specific BIN1 protein isoforms (isoforms 6, 9, 10 and 12) expressed by these cells. Except for isoform 6, these isoforms are characterized by the lack of a CLAP domain, and they appear to be expressed at higher level in microglia-like cells than in peripheral myeloid cells. The CLAP domain has been described to be involved in clathrin-mediated endocytosis and in the recruitment of dynamin. The absence of exon 7 and the CLAP domain in BIN1 isoforms expressed by microglia suggest that BIN1 may have a function during aging and AD that is unrelated to clathrin-mediated endocytosis. BIN1 has been implicated in phagocytosis in macrophages [43], and an increase of incidence of inflammation has been reported in BIN1 knock out mice [18], suggesting a possible anti-inflammatory function of BIN1. In addition, we show that BIN1 isoforms 10 and 12 are expressed in MDMi and not in peripheral monocytes, highlighting the existence of context-specific expression of BIN1 protein isoforms by microglia.

## Conclusions

Overall, using multiple approaches, our study has refined the cell-type specific expression patterns of the different BIN1 isoforms at the protein level. Our findings showed a strong association between BIN1 isoforms expressed by neurons/astrocytes and tangles that contributes to cognitive decline in AD, and our in situ studies refined these association to prioritize astrocytes as the target cell type. While these isoforms may be unrelated to the effect of the rs6733839 AD risk variant, they nonetheless represent a distinct contribution of this complex protein to AD, offering new insights into how BIN1 could be targeted or could serve as an outcome measure in AD studies.

## Supplementary information

**Additional file 1.**

**Additional file 2.**

**Additional file 3.**

**Additional file 4.**

**Additional file 5.**

**Additional file 6.**

## Data Availability

All data used and analyzed for the current study are available from the corresponding author on reasonable request.

## Abbreviations

AD: Alzheimer’s Disease
BIN1: Bridging integrator 1
PI: Phosphoinositide
CLAP: Clathrin and AP2
MBD: Myc-binding domain
SH3: Src homology 3
CNS: Central nervous system
DAB: Diaminobenzidine
ROS: Religious Orders Study
MAP: Memory and Aging Project
MCI: Mild cognitive impairment
PBMC: Peripheral blood mononuclear cells
MDMi: Monocyte Derived Microglia Like cells
DLPFC: Dorsolateral prefrontal cortex
SRM: Selected Reaction Monitoring
FDR: False Discovery Rate
