# Supplementary figures and images for: BIN1 protein isoforms are differentially expressed in astrocytes, neurons, and microglia: neuronal and astrocyte BIN1 are implicated in tau pathology
Source: Mol Neurodegener. 2020 Jul 29;15:44. doi: 10.1186/s13024-020-00387-3 (PMC7389646; doi:10.1186/s13024-020-00387-3)

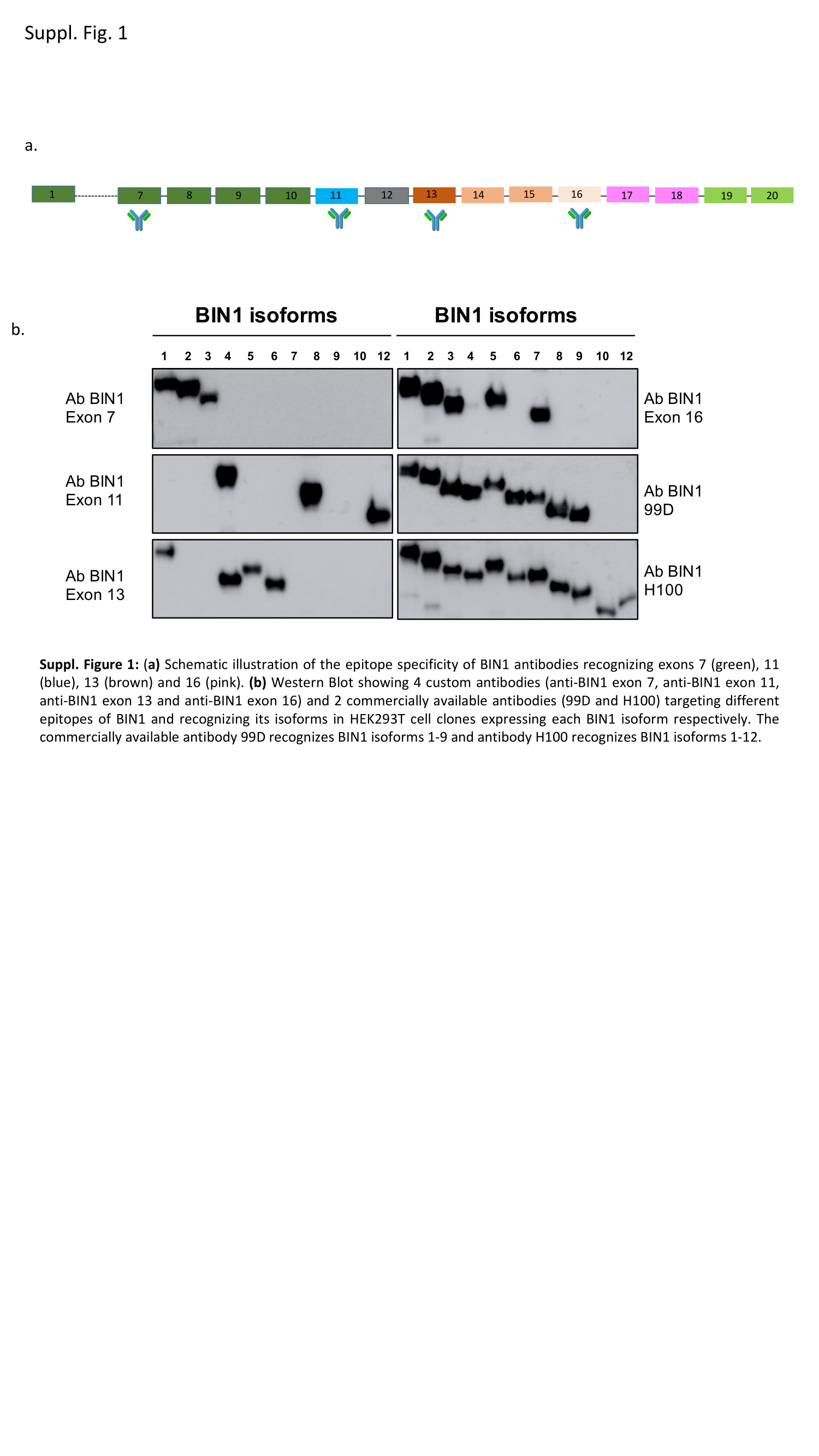

Supplement: Supplementary file 1 — Additional file 1. [file 13024_2020_387_MOESM1_ESM.tiff]

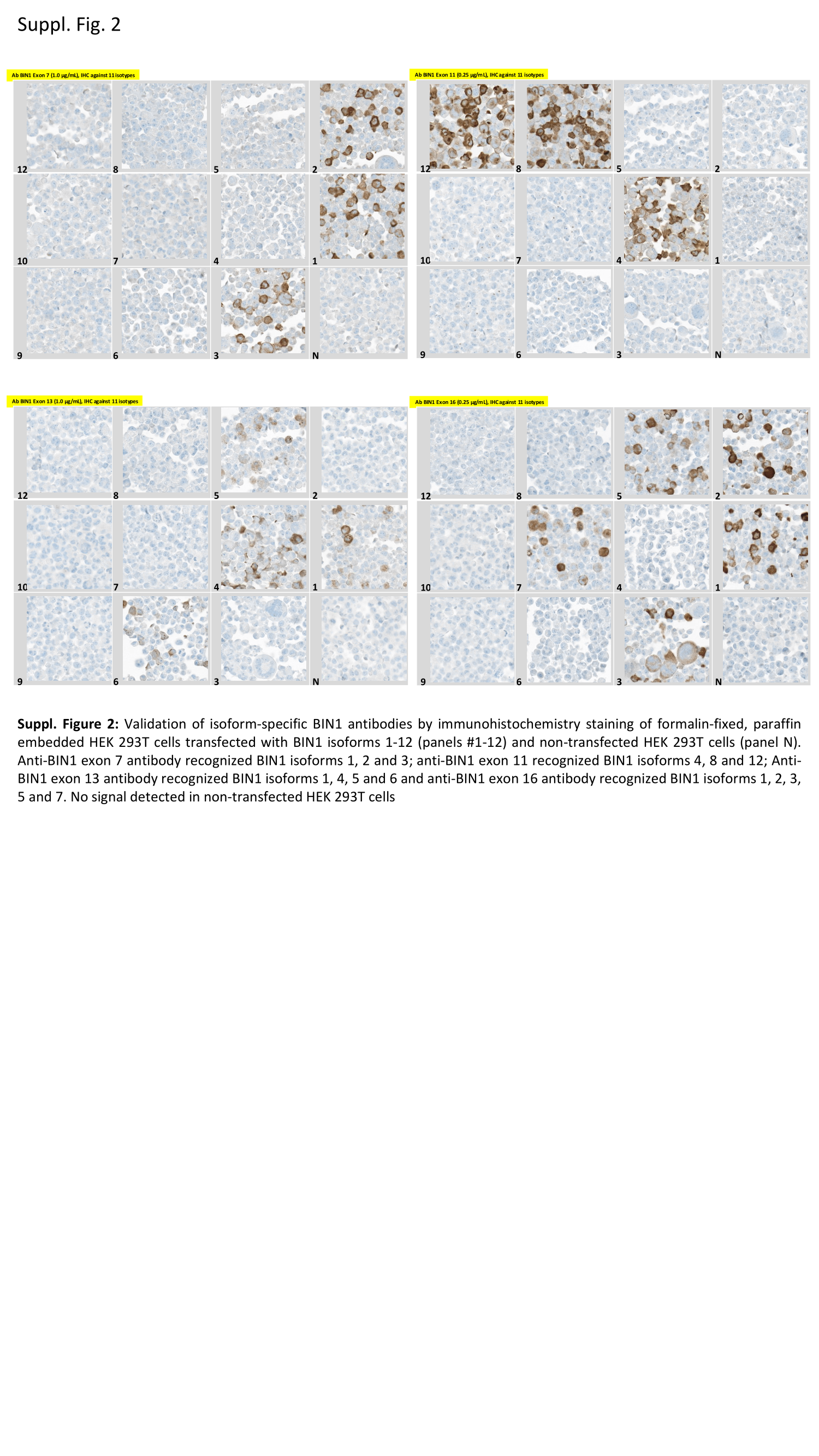

Supplement: Supplementary file 2 — Additional file 2. [file 13024_2020_387_MOESM2_ESM.tiff]

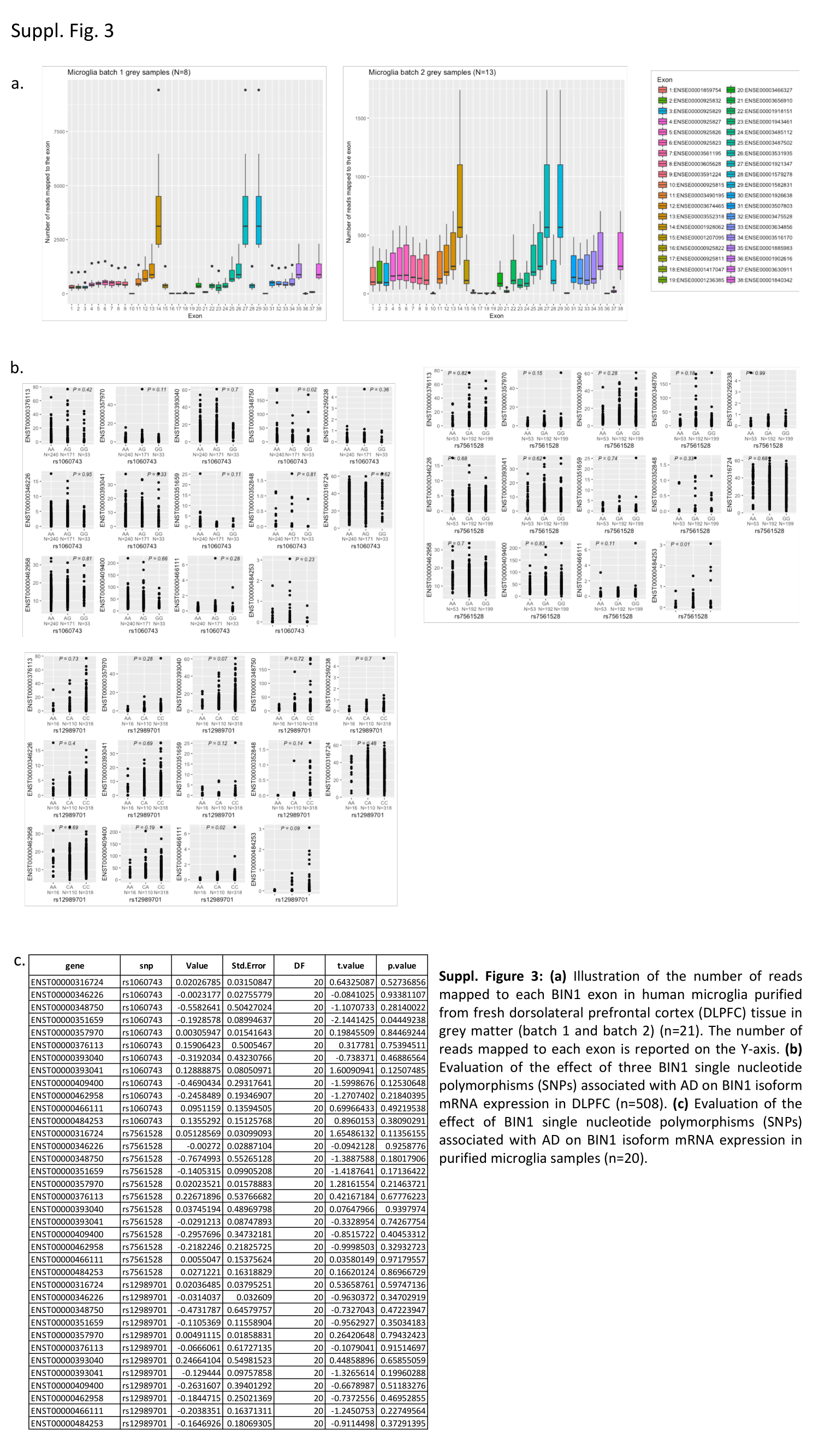

Supplement: Supplementary file 3 — Additional file 3. [file 13024_2020_387_MOESM3_ESM.tiff]

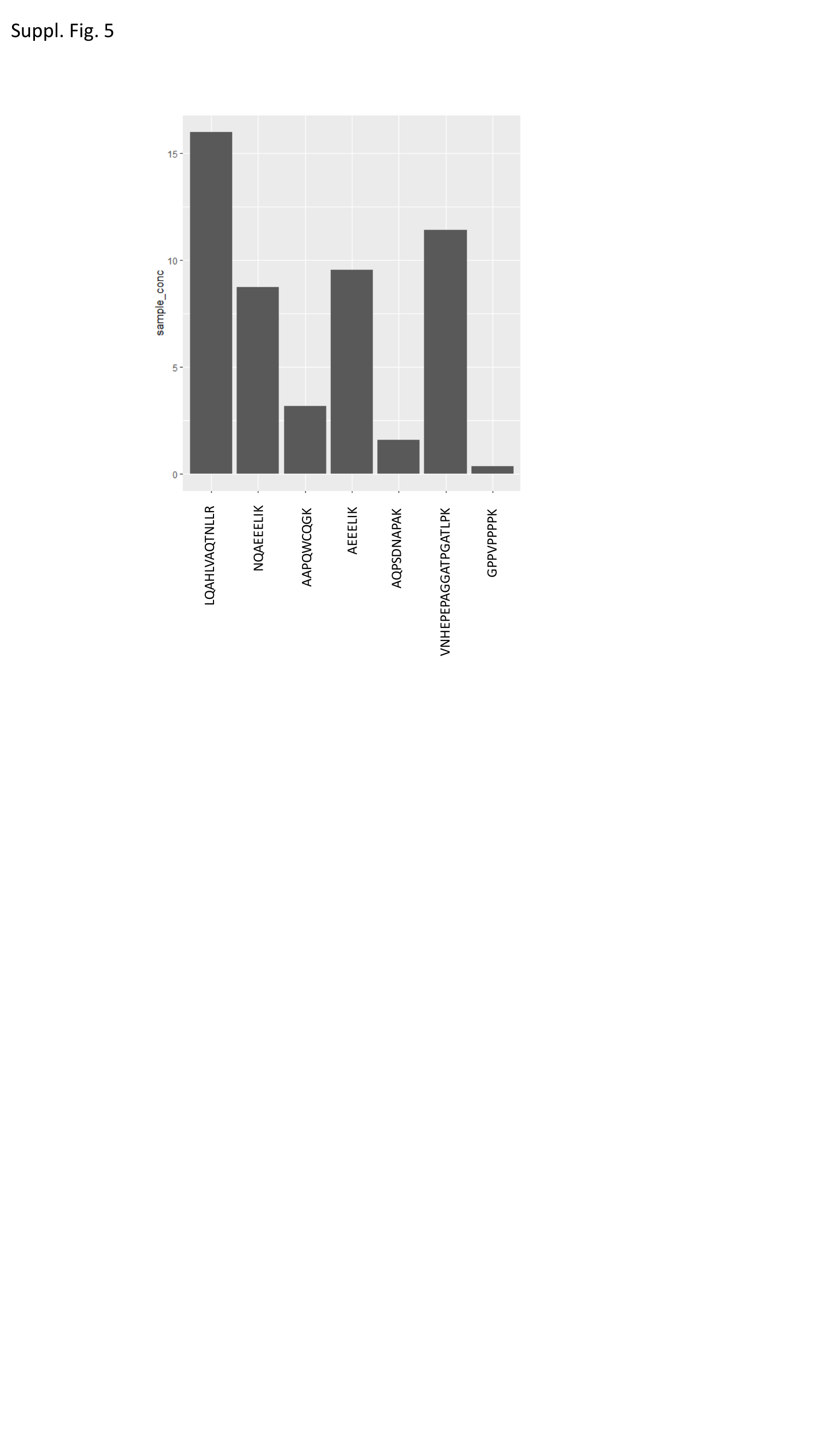

Supplement: Supplementary file 5 — Additional file 5. [file 13024_2020_387_MOESM5_ESM.tiff]
