# Supplementary material for: BIN1 protein isoforms are differentially expressed in astrocytes, neurons, and microglia: neuronal and astrocyte BIN1 are implicated in tau pathology
Source: Mol Neurodegener. 2020 Jul 29;15:44. doi: 10.1186/s13024-020-00387-3 (PMC7389646; doi:10.1186/s13024-020-00387-3)
Supplement: Supplementary file 6 — Additional file 6. [file 13024_2020_387_MOESM6_ESM.pdf]

Suppl. Table 1

a.

	cortical RNA-seq (n=508)
Male	38%
Female	62%
Pathological AD	58%
Clinical AD	38%
Age of death	88.4 +- 6.6

	SRM proteomics (n=1377)
Male	31.7%
Female	68.3%
Pathological AD	64.5%
Clinical AD	41.8%
Age of death	89.4 +- 6.5

b.

Gender	Age at death	Pathological Diagnosis	Cohort/Brain Bank
F	87	AD	MAP
F	101	AD	MAP
M	93	AD	MAP
F	99	AD	ROS
F	93	AD	MAP
F	96	AD	MAP
F	94	AD	MAP
F	98	AD	MAP
F	92	AD	MAP
F	91	AD	MAP
F	88	AD	MAP
F	95	AD	MAP
F	95	AD	MAP
F	96	AD	MAP
M	82	AD	MAP
F	96	AD	MAP
F	97	AD	ROS
F	94	non-AD	ROS
F	97	non-AD	MAP
M	87	non-AD	MAP
F	90	AD	MAP
F	61	AD	NYBB
M	61	AD	NYBB
M	78	AD	NYBB
F	75	AD	NYBB
M	79	AD	NYBB
F	65	AD	NYBB
F	69	AD	NYBB
F	73	AD	NYBB
M	67	AD	NYBB
F	68	non-AD	NYBB
M	67	non-AD	NYBB
F	85	non-AD	NYBB
M	81	non-AD	NYBB
F	82	non-AD	NYBB
F	62	non-AD	NYBB
M	64	non-AD	NYBB
M	82	non-AD	NYBB
M	65	non-AD	NYBB

Suppl. Table.1: (a) Demographic characteristics of the cortical RNA-seq samples (n=508) and SRM proteomics samples (n=1377). The AD pathology is defined using NIA-Reagan neuropathologic criteria (score 1-2) **(b) Description of subjects obtained from ROSMAP cohort and New York Brain Bank (NYBBB).** Human microglia RNA-seq and LC-MS/MS shotgun proteomics data were obtained from 21 ROSMAP subjects and all immunohistochemistry staining has been performed on 18 subjects from NYBB. The AD pathology is defined using NIA-Reagan neuropathologic criteria (score 1-2).

Suppl. Table 2

Peptide sequence	CONSeQuence Rank Score	exon
LQAHLVAQTNLLR	0.544	7
NQAEEELIK	0.267	7
AAPQWCQGK	0.255	7
AEEELIK	0.215	8
AQPSDNAPAK	0.236	10
VNHEPEPAGGATPGATLPK	0.899	12
GPPVPPPPK	0.085	13

Suppl. Table.2: Selection of 7 BIN1 peptides using the peptide detectability prediction tool CONSeQuence. The table presents the CONSeQuence rank scores for the 7 BIN1 peptides encoded by exons 7, 8, 10, 12 and 13.

Suppl. Table 3

Peptide sequence	exon	isoform
AGDVVLVIPFQNPEEQDEGWLMGV	15-16	all isoforms
LNQNLNDVLVGLEK	9	all isoforms
LVDQALLTMDTYLGQFPDIK	5	all isoforms
VGFYVNTFQSIAGLEENFHK	8	all isoforms
VNHEPEPAGGATPGATLPK	11	all isoforms
VQAQHDYTATDTDELQLK	15	all isoforms

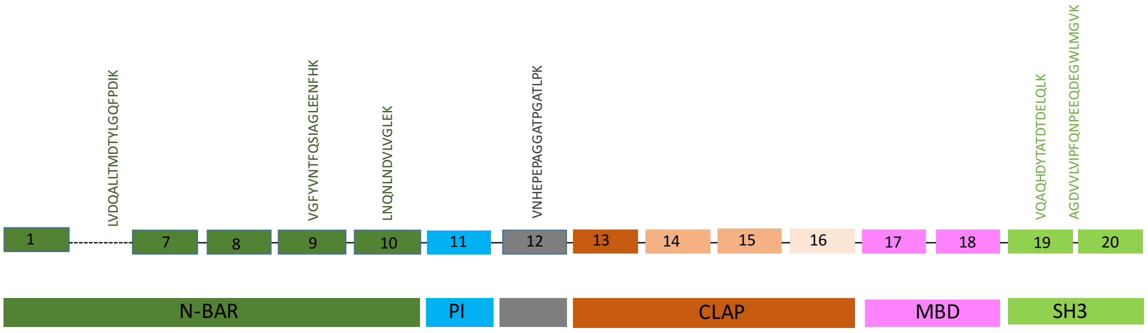

Suppl. Table.3: Detection of BIN1 peptides in purified human microglia. Peptide sequences for 6 BIN1 peptides encoded by exons 15-16, 9, 5, 8, 11 and 15, all expressed in all isoforms of BIN1 are detected in purified human microglia (n=2) in non-AD (NIA Reagan score=3) and AD subjects ((NIA Reagan score=2). The peptides have been selected based on their enrichment in microglia using the MaxQuant software. The peptide identification False Discovery Rate (FDR) has been set to 1%.

Suppl. Table 4

a.

BIN1 isoforms	ENST_gencode19	number of SNPs < 5.57E-5	number of SNPs < 0.05
1	ENST00000316724	0	73
2	ENST00000357970	0	6
3	ENST00000351659	0	194
4	ENST00000259238	0	3
5	ENST00000346226	0	0
6	ENST00000393040	0	65
7	ENST00000393041	0	127
8	ENST00000352848	N/A	N/A
9	ENST00000409400	0	60
10	ENST00000348750	0	82
12	ENST00000376113	0	57

b.

Id	Gencode_id	chr	position	Effective allele	a2	mac	Freq	Effect	Stdev	Z	*p*
rs1060743	ENST00000348750	2	127826533	A	G	262.96	0.73	-0.16	0.07	-2.25	0.02

Suppl. Table.4: Evaluation of the effect of BIN1 single nucleotide polymorphisms (SNPs) on BIN1 isoform mRNA expression in DLPFC. Expression levels for BIN1 isoforms are derived based on the RNA sequencing data from the DLPFC. The analysis adjusted for age at death, sex, and major ethnic principal components. (**a**) Evaluation of the effect of BIN1 SNPs on BIN1 isoform expression at the mRNA level in human DLPFC. The number of SNPs with Bonferroni corrected significance ($p<0.05/898=5.57 \times 10^{-5}$) and nominal significance ($p<0.05$) were presented. (**b)** Association between the AD-associated SNP rs1060743 and the expression level of isoform ENST00000348750 (isoform 10). Effect, Stderr, Z, and P represents beta estimate, its corresponding standard error, z statistic, and P value, respectively.

Suppl. Table 5

Peptides	*fdr* _AD dementia	*fdr* _Slope of Cognitive Decline	*fdr* _Residual Cog	*fdr* _Pathologic Diag.	*fdr* _Amyloid Burden	*fdr* _Tangle Burden
LQAHLVAQTNLLR	0.026007692	0.0002115	0.000780938	0.0002954	1.91E-05	1.89E-07
NQAEEELIK	0.001569167	6.98E-05	1.16E-05	6.84E-05	6.26E-08	2.34E-09
AAPQWCQGK	0.112397727	0.027375	0.026007692	0.00358575	0.052181818	0.00329921
AEEELIK	0.553875	0.523561644	0.290625	0.253584906	0.013923913	0.28254545
AQPSDNAPAK	0.770823529	0.544090909	0.318559322	0.318559322	0.01435	0.11239773
VNHEPEPAGGATPGATLPK	0.907644231	0.811774194	0.523561644	0.527837838	0.1579375	0.54759494
GPPVPPPPK	0.122266667	0.028965517	0.026911111	0.434538462	0.907644231	0.11239773

Suppl. Table.5: Association of BIN1 peptides with AD traits. The table presents the linear regression associations of 7 BIN1 peptides with clinical AD, slope of cognitive decline, pathological diagnosis of AD, amyloid and tangle burden (all values are presented here), and cognitive reserve (cognition adjusted for neuropathology) in DLPFC. Models were adjusted for age at death, sex and cohort (ROS or MAP).

Suppl. Table 6

Peptides	*fdr* _CAA	*fdr* _ci_num2_gct	*fdr* _ci_num2_mct	*fdr* _Lew Body	*fdr* _Hippocampal Sclerosis	*fdr* _TDP43 pathology
LQAHLVAQTNLLR	0.033396774	0.929	0.845585106	0.875357143	0.055897059	0.156382979
NQAEEELIK	0.033396774	0.774375	0.888787879	0.7425	0.16632	0.16632
AAPQWCQGK	0.070291667	0.875357143	0.771627907	0.875357143	0.38675	0.501338028
AEEELIK	0.544090909	0.907644231	0.807333333	0.697048193	0.656890244	0.501338028
AQPSDNAPAK	0.849947368	0.076263158	0.547594937	0.907644231	0.58462963	0.791629213
VNHEPEPAGGATPGATLPK	0.470149254	0.811774194	0.774375	0.397622951	0.811774194	0.486397059
GPPVPPPPK	0.09555	0.907644231	0.544090909	0.172941176	0.451818182	0.434538462

Suppl. Table.6: Association of BIN1 peptides with non-AD traits. The table presents the regression results of 7 BIN1 peptides with other pathology measures: CAA (Cerebral Amyloid Angiopathy), hippocampal sclerosis, Lewy Bodies, and the burden of TDP43 pathology. These models were adjusted for age of death, sex and cohort (ROS or MAP). No significant association has been detected between BIN1 peptides and the pathologies listed above at an FDR threshold of < 0.05.

Suppl. Table 7

Peptides	*fdr* _AD dementia	*fdr* _Slope of Cognitive Decline	*fdr* _Residual Cog	*fdr* _Pathologic Diag.	*fdr* _Amyloid Burden	*fdr* _Tangle Burden
LQAHLVAQTNLLR	0.833269231	0.159	0.2905	0.036	0.0105	0.02044875
NQAEEELIK	0.853915663	0.7940625	0.792105263	0.036	0.02044875	0.389454545
AAPQWCQGK	0.512195122	0.448636364	0.448636364	0.118363636	0.344842105	0.285352941
AEEELIK	0.920684211	0.853915663	0.823636364	0.448636364	0.792105263	0.9576
AQPSDNAPAK	0.448636364	0.777	0.719150943	0.862670455	0.823636364	0.495833333
VNHEPEPAGGATPGATLPK	0.823636364	0.823636364	0.823636364	0.63	0.890217391	0.853915663
GPPVPPPPK	0.823636364	0.525	0.495833333	0.512195122	0.7940625	0.814545455

Suppl. Table.7: Association of BIN1 peptides with AD pathologies after adjusting with cell proportion. The table presents the regression results of 7 BIN1 peptides with neuropathologies and cognitive characteristics (clinical AD, cognitive decline, cognitive reserve, pathological AD diagnosis, amyloid burden, and tangle burden), additionally adjusting for cell type proportion. Linear regressions were adjusted for: age at death, sex, and cohort (ROS or MAP), as well as myeloid proportion, neuron proportion, astrocyte proportion, oligodendrocyte proportion, and endothelial cell proportion as estimated using DSA (Digital Sorting Algorithm) with RNA-seq.

Suppl. Table 8

Peptides	*fdr* _Amyloid_adj	*fdr* _ Tangle_adj
AAPQWCQGK	0.33716667	0.02235333
LQAHLVAQTNLLR	0.04386667	0.00021175
NQAEEELIK	0.00749	0.0000728
AEEELIK	0.03885	0.971
AQPSDNAPAK	0.0525	0.6314
VNHEPEPAGGATPGATLPK	0.1918	0.971
GPPVPPPPK	0.492	0.056875

Suppl. Table.8: Association of BIN1 peptides encoded by exon 7 with tangle/amyloid burdens after adjusting with tangle/amyloid burdens. The tables present regressions results of BIN1 peptides encoded by exon 7 and tangle and amyloid burdens after adjustment for the other pathology, amyloid and tangle burdens, respectively. Linear regressions were also adjusted for age of death, sex, and cohort.

Suppl. Table 9

a.

Effect of BIN1 peptide on cognitive decline

Peptide	Beta	r2	*p*
**LQAHLVAQTNLLR**	0.0564	0.0148	4.63E-05
**NQAEEELIK**	0.0775	0.0179	7.34E-06
**AAPQWCQGK**	0.0287	0.00553	0.013
**AEEELIK**	-0.00473	0.00201	0.134
**AQPSDNAPAK**	-0.00497	0.00117	0.254
**VNHEPEPAGGATPGATLPK**	-0.00547	0.000736	0.365
**GPPVPPPPK**	0.0262	0.00315	0.0605

Effect of tangles on cognitive decline

Beta	r2	*p*
-0.0327	0.2	7.59E-67

b.

Effect of BIN1 peptide on cognitive decline after adjustment with tangle burden

Peptide	Beta	r2	*p*
**LQAHLVAQTNLLR**	0.029	0.00488	0.0202
**NQAEEELIK**	0.0377	0.00531	0.0153
**AAPQWCQGK**	0.0138	0.00163	0.18
**AEEELIK**	-0.0047	0.00256	0.0927
**AQPSDNAPAK**	-0.00404	0.000999	0.295
**VNHEPEPAGGATPGATLPK**	-0.0063	0.00126	0.238
**GPPVPPPPK**	0.00697	0.000285	0.575

c.

Effect of tangles on cognitive decline after adjustment with BIN1 peptide

Peptide	Beta	r2	*p*
**LQAHLVAQTNLLR**	-0.0332	0.197	1.59E-54
**NQAEEELIK**	-0.0331	0.196	3.22E-54
**AAPQWCQGK**	-0.0333	0.199	5.36E-55
**AEEELIK**	-0.0339	0.207	1.65E-57
**AQPSDNAPAK**	-0.0337	0.204	2.98E-56
**VNHEPEPAGGATPGATLPK**	-0.0339	0.207	1.99E-57
**GPPVPPPPK**	-0.0338	0.204	1.07E-56

Suppl. Table.9: Study of correlation between BIN1 peptide/APOE4 and cognitive decline. The tables presents the regression results of BIN1 peptides with cognitive decline. Linear regressions of 7 BIN1 peptides versus cognitive decline were performed, first without adjusting for tangle burden (**a**), and then adjusting for tangle burden (**b**). Regression of tangles on cognitive decline adjusting for BIN1 peptide is presented (**c**). Linear regressions of r^2s reported are partial r^2s.

Suppl. Table 10

SNP	Peptides	beta	se	tstat	pval	n	df
rs10498633	LQAHLVAQTNLLR	-0.0022	0.0114	-0.194	0.846	1018	5;1013
rs10498633	NQAEEELIK	-0.00343	0.00893	-0.384	0.701	1018	5;1013
rs10498633	AAPQWCQGK	-0.0111	0.0127	-0.876	0.381	1015	5;1010
rs10498633	AEEELIK	-0.0257	0.0505	-0.508	0.611	1018	5;1013
rs10498633	AQPSDNAPAK	0.00223	0.0368	0.0607	0.952	1012	5;1007
rs10498633	VNHEPEPAGGATPGATLPK	-0.0137	0.0265	-0.517	0.605	1017	5;1012
rs10498633	GPPVPPPPK	-0.0055	0.0113	-0.485	0.628	1018	5;1013
rs10792832	LQAHLVAQTNLLR	0.00533	0.01	0.531	0.596	1018	5;1013
rs10792832	NQAEEELIK	0.0147	0.00788	1.86	0.0631	1018	5;1013
rs10792832	AAPQWCQGK	-0.00608	0.0112	-0.542	0.588	1015	5;1010
rs10792832	AEEELIK	-0.0803	0.0446	-1.8	0.0722	1018	5;1013
rs10792832	AQPSDNAPAK	-0.0128	0.0325	-0.395	0.693	1012	5;1007
rs10792832	VNHEPEPAGGATPGATLPK	-0.0286	0.0234	-1.22	0.222	1017	5;1012
rs10792832	GPPVPPPPK	-0.0152	0.01	-1.52	0.129	1018	5;1013
rs10838725	LQAHLVAQTNLLR	-0.00383	0.0105	-0.364	0.716	1018	5;1013
rs10838725	NQAEEELIK	-0.0026	0.00827	-0.314	0.753	1018	5;1013
rs10838725	AAPQWCQGK	-0.0124	0.0117	-1.05	0.293	1015	5;1010
rs10838725	AEEELIK	0.0197	0.0468	0.42	0.675	1018	5;1013
rs10838725	AQPSDNAPAK	-0.0263	0.034	-0.773	0.44	1012	5;1007
rs10838725	VNHEPEPAGGATPGATLPK	0.0304	0.0245	1.24	0.216	1017	5;1012
rs10838725	GPPVPPPPK	0.00472	0.0105	0.449	0.653	1018	5;1013
rs10948363	LQAHLVAQTNLLR	-0.0192	0.0109	-1.76	0.0792	1018	5;1013
rs10948363	NQAEEELIK	-0.0196	0.0086	-2.27	0.0231	1018	5;1013
rs10948363	AAPQWCQGK	-0.0136	0.0122	-1.11	0.268	1015	5;1010
rs10948363	AEEELIK	0.0223	0.0488	0.456	0.649	1018	5;1013
rs10948363	AQPSDNAPAK	0.00882	0.0356	0.248	0.804	1012	5;1007
rs10948363	VNHEPEPAGGATPGATLPK	0.0139	0.0256	0.543	0.587	1017	5;1012
rs10948363	GPPVPPPPK	-0.00565	0.0109	-0.517	0.606	1018	5;1013
rs11218343	LQAHLVAQTNLLR	-0.00627	0.0267	-0.234	0.815	1018	5;1013
rs11218343	NQAEEELIK	-0.00969	0.021	-0.461	0.645	1018	5;1013
rs11218343	AAPQWCQGK	-0.0192	0.0298	-0.643	0.52	1015	5;1010
rs11218343	AEEELIK	0.0126	0.119	0.106	0.916	1018	5;1013
rs11218343	AQPSDNAPAK	-0.0237	0.087	-0.273	0.785	1012	5;1007
rs11218343	VNHEPEPAGGATPGATLPK	0.0106	0.0624	0.17	0.865	1017	5;1012
rs11218343	GPPVPPPPK	-0.000202	0.0267	-0.00758	0.994	1018	5;1013
rs1171812	LQAHLVAQTNLLR	0.00831	0.0095	0.875	0.382	1018	5;1013
rs1171812	NQAEEELIK	0.0132	0.00747	1.77	0.0776	1018	5;1013
rs1171812	AAPQWCQGK	0.0216	0.0106	2.04	0.0419	1015	5;1010
rs1171812	AEEELIK	-0.0295	0.0423	-0.697	0.486	1018	5;1013
rs1171812	AQPSDNAPAK	-0.00562	0.0308	-0.182	0.855	1012	5;1007
rs1171812	VNHEPEPAGGATPGATLPK	-0.0131	0.0222	-0.589	0.556	1017	5;1012
rs1171812	GPPVPPPPK	0.00985	0.00949	1.04	0.3	1018	5;1013
rs11771145	LQAHLVAQTNLLR	0.00532	0.0114	0.465	0.642	1018	5;1013
rs11771145	NQAEEELIK	0.00694	0.009	0.771	0.441	1018	5;1013
rs11771145	AAPQWCQGK	0.00109	0.0128	0.0849	0.932	1015	5;1010
rs11771145	AEEELIK	-0.0856	0.0509	-1.68	0.093	1018	5;1013
rs11771145	AQPSDNAPAK	-0.037	0.0371	-0.996	0.319	1012	5;1007
rs11771145	VNHEPEPAGGATPGATLPK	-0.0396	0.0267	-1.49	0.138	1017	5;1012
rs11771145	GPPVPPPPK	0.00982	0.0114	0.86	0.39	1018	5;1013
rs12444183	LQAHLVAQTNLLR	0.00205	0.01	0.205	0.838	1018	5;1013
rs12444183	NQAEEELIK	-0.0043	0.00789	-0.545	0.586	1018	5;1013
rs12444183	AAPQWCQGK	-0.0105	0.0112	-0.935	0.35	1015	5;1010
rs12444183	AEEELIK	0.0392	0.0447	0.878	0.38	1018	5;1013
rs12444183	AQPSDNAPAK	-0.0285	0.0325	-0.877	0.381	1012	5;1007
rs12444183	VNHEPEPAGGATPGATLPK	0.0213	0.0234	0.909	0.364	1017	5;1012
rs12444183	GPPVPPPPK	-0.000817	0.01	-0.0815	0.935	1018	5;1013
rs1476679	LQAHLVAQTNLLR	-0.0172	0.0105	-1.64	0.1	1018	5;1013
rs1476679	NQAEEELIK	0.00479	0.00824	0.581	0.561	1018	5;1013
rs1476679	AAPQWCQGK	-0.00233	0.0117	-0.199	0.842	1015	5;1010
rs1476679	AEEELIK	-0.0192	0.0466	-0.411	0.681	1018	5;1013
rs1476679	AQPSDNAPAK	-0.0504	0.0339	-1.49	0.138	1012	5;1007
rs1476679	VNHEPEPAGGATPGATLPK	-0.017	0.0245	-0.695	0.487	1017	5;1012
rs1476679	GPPVPPPPK	0.000948	0.0105	0.0907	0.928	1018	5;1013
rs17125944	LQAHLVAQTNLLR	-0.016	0.0169	-0.95	0.342	1018	5;1013
rs17125944	NQAEEELIK	-0.00259	0.0133	-0.195	0.845	1018	5;1013
rs17125944	AAPQWCQGK	-0.00728	0.0188	-0.387	0.699	1015	5;1010
rs17125944	AEEELIK	0.0515	0.0751	0.686	0.493	1018	5;1013
rs17125944	AQPSDNAPAK	0.0141	0.0547	0.258	0.797	1012	5;1007
rs17125944	VNHEPEPAGGATPGATLPK	0.00547	0.0393	0.139	0.89	1017	5;1012
rs17125944	GPPVPPPPK	0.00316	0.0168	0.188	0.851	1018	5;1013
rs190982	LQAHLVAQTNLLR	0.00709	0.0106	0.668	0.504	1018	5;1013
rs190982	NQAEEELIK	-0.00208	0.00835	-0.249	0.804	1018	5;1013
rs190982	AAPQWCQGK	0.00266	0.0119	0.224	0.823	1015	5;1010
rs190982	AEEELIK	-0.0298	0.0473	-0.63	0.529	1018	5;1013
rs190982	AQPSDNAPAK	-0.0178	0.0344	-0.518	0.605	1012	5;1007
rs190982	VNHEPEPAGGATPGATLPK	-0.0136	0.0248	-0.551	0.582	1017	5;1012
rs190982	GPPVPPPPK	0.00721	0.0106	0.68	0.497	1018	5;1013
rs2718058	LQAHLVAQTNLLR	-0.0198	0.0105	-1.89	0.0586	1018	5;1013
rs2718058	NQAEEELIK	-0.00316	0.00826	-0.382	0.702	1018	5;1013
rs2718058	AAPQWCQGK	-0.000266	0.0118	-0.0227	0.982	1015	5;1010
rs2718058	AEEELIK	-0.0293	0.0468	-0.626	0.531	1018	5;1013
rs2718058	AQPSDNAPAK	-0.0104	0.0341	-0.304	0.761	1012	5;1007
rs2718058	VNHEPEPAGGATPGATLPK	-0.0157	0.0245	-0.643	0.521	1017	5;1012
rs2718058	GPPVPPPPK	-0.00641	0.0105	-0.612	0.541	1018	5;1013
rs28834970	LQAHLVAQTNLLR	-0.0128	0.0101	-1.26	0.207	1018	5;1013
rs28834970	NQAEEELIK	0.00872	0.00796	1.1	0.273	1018	5;1013
rs28834970	AAPQWCQGK	0.0234	0.0113	2.08	0.0379	1015	5;1010
rs28834970	AEEELIK	0.0317	0.0451	0.704	0.481	1018	5;1013
rs28834970	AQPSDNAPAK	0.0325	0.0327	0.992	0.322	1012	5;1007
rs28834970	VNHEPEPAGGATPGATLPK	0.0175	0.0236	0.742	0.458	1017	5;1012
rs28834970	GPPVPPPPK	0.0196	0.0101	1.94	0.0522	1018	5;1013

SNP	Peptides	beta	se	tstat	pval	n	df
rs35349669	LQAHLVAQTNLLR	-0.00368	0.0107	-0.343	0.732	1018	5;1013
rs35349669	NQAEEELIK	-0.00533	0.00844	-0.631	0.528	1018	5;1013
rs35349669	AAPQWCQGK	0.000858	0.012	0.0716	0.943	1015	5;1010
rs35349669	AEEELIK	0.0186	0.0478	0.389	0.697	1018	5;1013
rs35349669	AQPSDNAPAK	0.000725	0.0348	0.0208	0.983	1012	5;1007
rs35349669	VNHEPEPAGGATPGATLPK	0.00949	0.025	0.379	0.705	1017	5;1012
rs35349669	GPPVPPPPK	0.00798	0.0107	0.745	0.457	1018	5;1013
rs3865444	LQAHLVAQTNLLR	-0.00215	0.0105	-0.206	0.837	1018	5;1013
rs3865444	NQAEEELIK	-0.00322	0.00823	-0.391	0.696	1018	5;1013
rs3865444	AAPQWCQGK	-0.0178	0.0117	-1.53	0.127	1015	5;1010
rs3865444	AEEELIK	-0.0246	0.0466	-0.528	0.598	1018	5;1013
rs3865444	AQPSDNAPAK	0.0219	0.0339	0.647	0.518	1012	5;1007
rs3865444	VNHEPEPAGGATPGATLPK	-0.00685	0.0244	-0.281	0.779	1017	5;1012
rs3865444	GPPVPPPPK	-0.0191	0.0104	-1.83	0.0676	1018	5;1013
rs4147929	LQAHLVAQTNLLR	-0.0187	0.0141	-1.32	0.186	1018	5;1013
rs4147929	NQAEEELIK	-0.000184	0.0111	-0.0166	0.987	1018	5;1013
rs4147929	AAPQWCQGK	-0.00561	0.0158	-0.355	0.722	1015	5;1010
rs4147929	AEEELIK	0.032	0.0629	0.508	0.612	1018	5;1013
rs4147929	AQPSDNAPAK	0.052	0.0457	1.14	0.256	1012	5;1007
rs4147929	VNHEPEPAGGATPGATLPK	0.0484	0.033	1.47	0.142	1017	5;1012
rs4147929	GPPVPPPPK	-0.00465	0.0141	-0.329	0.742	1018	5;1013
rs442495	LQAHLVAQTNLLR	-0.00222	0.0105	-0.212	0.832	1018	5;1013
rs442495	NQAEEELIK	-0.00994	0.00822	-1.21	0.227	1018	5;1013
rs442495	AAPQWCQGK	-0.0241	0.0117	-2.07	0.0388	1015	5;1010
rs442495	AEEELIK	0.0153	0.0466	0.328	0.743	1018	5;1013
rs442495	AQPSDNAPAK	-0.0106	0.0339	-0.313	0.754	1012	5;1007
rs442495	VNHEPEPAGGATPGATLPK	0.00542	0.0244	0.222	0.824	1017	5;1012
rs442495	GPPVPPPPK	-0.0144	0.0104	-1.38	0.169	1018	5;1013
rs4575098	LQAHLVAQTNLLR	-0.00354	0.0119	-0.297	0.767	1018	5;1013
rs4575098	NQAEEELIK	0.00227	0.00938	0.241	0.809	1018	5;1013
rs4575098	AAPQWCQGK	0.015	0.0133	1.12	0.261	1015	5;1010
rs4575098	AEEELIK	-0.0148	0.0531	-0.278	0.781	1018	5;1013
rs4575098	AQPSDNAPAK	0.0371	0.0386	0.96	0.337	1012	5;1007
rs4575098	VNHEPEPAGGATPGATLPK	-0.0191	0.0278	-0.687	0.492	1017	5;1012
rs4575098	GPPVPPPPK	-0.000825	0.0119	-0.0693	0.945	1018	5;1013
rs6448453	LQAHLVAQTNLLR	0.0274	0.0108	2.53	0.0115	1018	5;1013
rs6448453	NQAEEELIK	0.00999	0.00852	1.17	0.241	1018	5;1013
rs6448453	AAPQWCQGK	0.00987	0.0121	0.815	0.415	1015	5;1010
rs6448453	AEEELIK	-0.0102	0.0483	-0.211	0.833	1018	5;1013
rs6448453	AQPSDNAPAK	0.0111	0.0351	0.317	0.751	1012	5;1007
rs6448453	VNHEPEPAGGATPGATLPK	-0.00807	0.0253	-0.319	0.75	1017	5;1012
rs6448453	GPPVPPPPK	0.00233	0.0108	0.215	0.829	1018	5;1013
rs6504163	LQAHLVAQTNLLR	0.00948	0.0117	0.811	0.418	1018	5;1013
rs6504163	NQAEEELIK	0.0145	0.00918	1.58	0.115	1018	5;1013
rs6504163	AAPQWCQGK	0.0136	0.0131	1.04	0.297	1015	5;1010
rs6504163	AEEELIK	0.0215	0.0521	0.413	0.68	1018	5;1013
rs6504163	AQPSDNAPAK	-0.0503	0.0379	-1.33	0.185	1012	5;1007
rs6504163	VNHEPEPAGGATPGATLPK	0.00355	0.0273	0.13	0.897	1017	5;1012
rs6504163	GPPVPPPPK	-0.00336	0.0117	-0.288	0.774	1018	5;1013
rs6656401	LQAHLVAQTNLLR	0.0149	0.0127	1.17	0.241	1018	5;1013
rs6656401	NQAEEELIK	0.0107	0.00997	1.07	0.283	1018	5;1013
rs6656401	AAPQWCQGK	0.0218	0.0142	1.54	0.124	1015	5;1010
rs6656401	AEEELIK	-0.0426	0.0565	-0.755	0.45	1018	5;1013
rs6656401	AQPSDNAPAK	-0.0377	0.0413	-0.912	0.362	1012	5;1007
rs6656401	VNHEPEPAGGATPGATLPK	-0.0185	0.0296	-0.624	0.533	1017	5;1012
rs6656401	GPPVPPPPK	0.0157	0.0127	1.24	0.215	1018	5;1013
rs6733839	LQAHLVAQTNLLR	0.0155	0.0125	1.24	0.217	1018	5;1013
rs6733839	NQAEEELIK	-0.0139	0.00984	-1.41	0.159	1018	5;1013
rs6733839	AAPQWCQGK	-0.0378	0.0139	-2.72	0.00674	1015	5;1010
rs6733839	AEEELIK	0.0393	0.0558	0.705	0.481	1018	5;1013
rs6733839	AQPSDNAPAK	0.0164	0.0407	0.402	0.688	1012	5;1007
rs6733839	VNHEPEPAGGATPGATLPK	0.0281	0.0292	0.964	0.336	1017	5;1012
rs6733839	GPPVPPPPK	-0.00985	0.0125	-0.788	0.431	1018	5;1013
rs7274581	LQAHLVAQTNLLR	0.00807	0.0177	0.456	0.648	1018	5;1013
rs7274581	NQAEEELIK	0.0132	0.0139	0.948	0.344	1018	5;1013
rs7274581	AAPQWCQGK	0.00147	0.0197	0.0743	0.941	1015	5;1010
rs7274581	AEEELIK	-0.111	0.0786	-1.41	0.159	1018	5;1013
rs7274581	AQPSDNAPAK	-0.0721	0.0575	-1.25	0.21	1012	5;1007
rs7274581	VNHEPEPAGGATPGATLPK	-0.0748	0.0413	-1.81	0.0702	1017	5;1012
rs7274581	GPPVPPPPK	-0.0151	0.0176	-0.855	0.393	1018	5;1013
rs9271192	LQAHLVAQTNLLR	0.0159	0.0107	1.48	0.139	1018	5;1013
rs9271192	NQAEEELIK	0.00598	0.00844	0.709	0.478	1018	5;1013
rs9271192	AAPQWCQGK	0.0116	0.012	0.967	0.334	1015	5;1010
rs9271192	AEEELIK	-0.041	0.0478	-0.859	0.39	1018	5;1013
rs9271192	AQPSDNAPAK	0.0389	0.0347	1.12	0.263	1012	5;1007
rs9271192	VNHEPEPAGGATPGATLPK	-0.0246	0.025	-0.984	0.325	1017	5;1012
rs9271192	GPPVPPPPK	0.00847	0.0107	0.791	0.429	1018	5;1013
rs9331896	LQAHLVAQTNLLR	-0.0196	0.0101	-1.94	0.0529	1018	5;1013
rs9331896	NQAEEELIK	-0.00154	0.00798	-0.193	0.847	1018	5;1013
rs9331896	AAPQWCQGK	0.00139	0.0113	0.123	0.902	1015	5;1010
rs9331896	AEEELIK	0.0425	0.0452	0.94	0.347	1018	5;1013
rs9331896	AQPSDNAPAK	0.115	0.0327	3.51	0.000475	1012	5;1007
rs9331896	VNHEPEPAGGATPGATLPK	0.0252	0.0237	1.06	0.288	1017	5;1012
rs9331896	GPPVPPPPK	0.00417	0.0101	0.412	0.681	1018	5;1013
rs983392	LQAHLVAQTNLLR	0.0124	0.0097	1.28	0.201	1018	5;1013
rs983392	NQAEEELIK	0.00558	0.00764	0.731	0.465	1018	5;1013
rs983392	AAPQWCQGK	0.0115	0.0108	1.06	0.287	1015	5;1010
rs983392	AEEELIK	0.0094	0.0432	0.217	0.828	1018	5;1013
rs983392	AQPSDNAPAK	-0.00949	0.0314	-0.302	0.763	1012	5;1007
rs983392	VNHEPEPAGGATPGATLPK	-0.000508	0.0227	-0.0224	0.982	1017	5;1012
rs983392	GPPVPPPPK	-0.00196	0.00969	-0.202	0.84	1018	5;1013

Suppl. Table.10: Study of the effect of AD susceptibility variants on BIN1 peptides in DLPFC. Linear regressions of BIN1 peptides versus genome-wide significant IGAP SNPs (Lambert *et al.*,) and Marioni family history of Alzheimer's SNPs (Marioni *et al.*,) were performed, adjusting for major ethnical principal components.

Suppl. Table 11

outcome	Peptides	beta	se	tstat	pval	n	fdr
tangles_sqrt	LQAHLVAQTNLLR	-0.8	0.243	-3.29	0.0011	430	0.0077
tangles_sqrt	NQAEEELIK	-0.495	0.316	-1.57	0.118	430	0.34533333
tangles_sqrt	AAPQWCQGK	-0.33	0.227	-1.45	0.148	430	0.34533333
tangles_sqrt	AEEELIK	0.00454	0.0574	0.0791	0.937	430	0.937
tangles_sqrt	AQPSDNAPAK	-0.051	0.0799	-0.639	0.523	430	0.91525
tangles_sqrt	VNHEPEPAGGATPGATLPK	-0.0294	0.108	-0.271	0.786	430	0.917
tangles_sqrt	GPPVPPPPK	-0.128	0.306	-0.419	0.675	430	0.917

Suppl. Table.11: Association of the BIN1 peptide LQAHLVAQTNLLR encoded by exon 7 with tangle burden. Regression results of the BIN1 peptide LQAHLVAQTNLLR encoded by exon 7 with tangle burden, adjusting for age at death, sex, as well as miRNA, and DLPFC modules (derived from RNA-seq), which were previously shown to be associated with tangle burden in the ROS and MAP cohorts.

Suppl. Table 12

Peptides	beta	se	tstat	pval	n	fdr
LQAHLVAQTNLLR	-0.000237	0.00021	-1.13	0.261	579	0.7175
NQAEEELIK	-0.000182	0.000156	-1.17	0.242	579	0.7175
AAPQWCQGK	-0.000325	0.000181	-1.8	0.0725	579	0.7175
AEEELIK	0.000753	0.000914	0.824	0.41	579	0.7175
AQPSDNAPAK	0.000249	0.00068	0.367	0.714	578	0.768923077
VNHEPEPAGGATPGATLPK	0.000208	0.000474	0.438	0.661	578	0.768923077
GPPVPPPPK	-0.000201	0.000162	-1.24	0.215	579	0.7175
LQAHLVAQTNLLR	-5.45E-05	0.00021	-0.26	0.795	579	0.795
NQAEEELIK	-0.000107	0.000156	-0.683	0.495	579	0.768923077
AAPQWCQGK	-0.000261	0.000184	-1.42	0.155	579	0.7175
AEEELIK	0.000786	0.000934	0.842	0.4	579	0.7175
AQPSDNAPAK	0.000271	0.000694	0.39	0.697	578	0.768923077
VNHEPEPAGGATPGATLPK	0.00026	0.000484	0.537	0.592	578	0.768923077
GPPVPPPPK	-0.000164	0.000166	-0.988	0.324	579	0.7175

Suppl. Table.12: Study of association between Tau-related epigenomic alterations and BIN1 peptides. Linear regressions of BIN1 peptides versus ETES (Epigenome Tau Effect Score) (a single score summarizing tau-related changes due to H3K9ac genome-wide) were performed, adjusting for age at death, and sex. No association between Tau-related epigenomic alterations and BIN1 peptides was detected.
